# Rate-dependent effects of lidocaine on cardiac dynamics: Development and analysis of a low-dimensional drug-channel interaction model

**DOI:** 10.1371/journal.pcbi.1009145

**Published:** 2021-06-29

**Authors:** Steffen S. Docken, Colleen E. Clancy, Timothy J. Lewis

**Affiliations:** 1 Department of Mathematics, University of California Davis, Davis, California, United States of America; 2 Department of Physiology and Membrane Biology, School of Medicine, University of California Davis, Davis, California, United States of America; University of Virginia, UNITED STATES

## Abstract

State-dependent sodium channel blockers are often prescribed to treat cardiac arrhythmias, but many sodium channel blockers are known to have pro-arrhythmic side effects. While the anti and proarrhythmic potential of a sodium channel blocker is thought to depend on the characteristics of its rate-dependent block, the mechanisms linking these two attributes are unclear. Furthermore, how specific properties of rate-dependent block arise from the binding kinetics of a particular drug is poorly understood. Here, we examine the rate-dependent effects of the sodium channel blocker lidocaine by constructing and analyzing a novel drug-channel interaction model. First, we identify the predominant mode of lidocaine binding in a 24 variable Markov model for lidocaine-sodium channel interaction by Moreno et al. Specifically, we find that (1) the vast majority of lidocaine bound to sodium channels is in the neutral form, i.e., the binding of charged lidocaine to sodium channels is negligible, and (2) neutral lidocaine binds almost exclusively to inactivated channels and, upon binding, immobilizes channels in the inactivated state. We then develop a novel 3-variable lidocaine-sodium channel interaction model that incorporates only the predominant mode of drug binding. Our low-dimensional model replicates an extensive amount of the voltage-clamp data used to parameterize the Moreno et al. model. Furthermore, the effects of lidocaine on action potential upstroke velocity and conduction velocity in our model are similar to those predicted by the Moreno et al. model. By exploiting the low-dimensionality of our model, we derive an algebraic expression for level of rate-dependent block as a function of pacing frequency, restitution properties, diastolic and plateau potentials, and drug binding rate constants. Our model predicts that the level of rate-dependent block is sensitive to alterations in restitution properties and increases in diastolic potential, but it is insensitive to variations in the shape of the action potential waveform and lidocaine binding rates.

## Author summary

Cardiac arrhythmias are often treated with drugs that block and alter the kinetics of membrane sodium channels. However, different drugs interact with sodium channels in different ways, and the complexity of the drug-channel interactions makes it difficult to predict whether a particular sodium channel blocker will reduce or increase the probability of cardiac arrhythmias. Here, we characterize the binding kinetics and effects on electrical signal propagation of the antiarrhythmic drug lidocaine, which is an archetypical example of a safe sodium channel blocker. Through analysis of a high-dimensional biophysically-detailed model of lidocaine-sodium channel interaction, we identify the predominant lidocaine binding pathway. We then incorporate only the key features of the predominant binding pathway into a novel low-dimensional model of lidocaine-sodium channel interaction. Our analysis of the low-dimensional model characterizes how the key binding properties of lidocaine affect electrical signal generation and propagation in the heart, and therefore our results are a step towards understanding the features that differentiate pro- and antiarrhythmic sodium channel blockers.

## 1. Introduction

Class I antiarrhythmic drugs bind to and block the fast sodium (Na^+^) channels that are responsible for the upstroke and propagation of the cardiac action potential. Na^+^ channel blockers are thought to exert their antiarrhythmic effects by reducing the excitability of cardiac tissue in a rate-dependent manner, thereby preventing aberrant spontaneous action potentials. However, blocking the Na^+^ current also slows action potential conduction, which can facilitate the onset of reentrant arrhythmias, especially at high heart rates [1,2]. Indeed, many Na^+^ channel blockers that are prescribed as antiarrhythmic drugs, such as flecainide, can increase the propensity of ventricular arrhythmias [3,4], whereas other Na^+^ channel blockers, such as lidocaine, are considered to be safe [5]. The mechanisms that make certain Na^+^ channel blockers antiarrhythmic and others pro-arrhythmic remain unclear.

To determine the arrhythmic potential of Na^+^ channel blockers, biophysically detailed models of Na^+^ channel-drug interactions have been constructed using a detailed Markov model framework [4,6–8]. Such models accurately reproduce voltage-clamp data that characterize the modulatory effects of drugs on channel kinetics [9–13], but come at the cost of being complex, consisting of up to 26 dynamic variables. While these high-dimensional models have been able to help determine if specific drugs prevent or exacerbate arrhythmia, their complexity impedes the ability to identify the fundamental features of drug-channel interactions that lead to pro-arrhythmic effects. This in turn makes it difficult to generalize knowledge of well-studied Na^+^ channel blockers, such as lidocaine or flecainide, to new drugs. Low dimensional or “minimal” models that contain only the key features of drug-ion channel interactions can be advantageous in identifying the fundamental mechanisms that underlie the rate-dependent block and resulting pro- or antiarrhythmic effects of some Na^+^ channel blockers, as such mechanisms would be brought to the foreground due to the simplicity of such models.

In this paper, we construct and analyze a novel low-dimensional lidocaine-Na^+^ channel interaction model that consists of 3 dynamic variables. We first analyze a detailed Markov model for lidocaine-Na^+^ channel interaction introduced by Moreno et al. [4] and identify the predominant mode of binding that defines lidocaine’s effects on the Na^+^ current. We then base the structure of our low-dimensional model on these essential features. The low-dimensional model is fit to voltage-clamp data that was used to parameterize the Moreno et al. model. We observe that our low-dimensional model and the high-dimensional Moreno et al. model reproduce the experimental data to a similar degree for physiological conditions. Additionally, we show that our model and the Moreno et al. model predict similar rate-dependent effects on action potential upstroke and conduction velocity. Finally, we utilize the simple structure of our low-dimensional model to understand the rate-dependent effects of lidocaine and how these effects are influenced by various physiological properties (e.g., the action potential duration restitution curve and the diastolic transmembrane potential).

## 2. Models

Cardiac Na^+^ channels are generally modeled as variable Ohmic resistors. The Na^+^ current is the product of the Na^+^ conductance (${\bar{g}}_{Na}f_{open}$) and the driving force (*V*−*E*_*Na*_),

$$I_{Na}={\bar{g}}_{Na}f_{open}(V-E_{Na}),$$ (1)

where *V* is the transmembrane potential, *E*_*Na*_ is the Na^+^ reversal potential, ${\bar{g}}_{Na}$ is the maximal conductance (i.e., the conductance when all channels are open), and *f*_*open*_ is the fraction of channels that are in an open and unblocked state. In this section, we describe and compare two mathematical models for the dynamics of *f*_*open*_ in the presence of lidocaine: A detailed Markov model by Moreno et al. [4], and a novel low-dimensional model with generalized Hodgkin-Huxley formalism that we develop here.

### 2.1. The Moreno et al. model: A detailed Markov model

**Fig 1:**
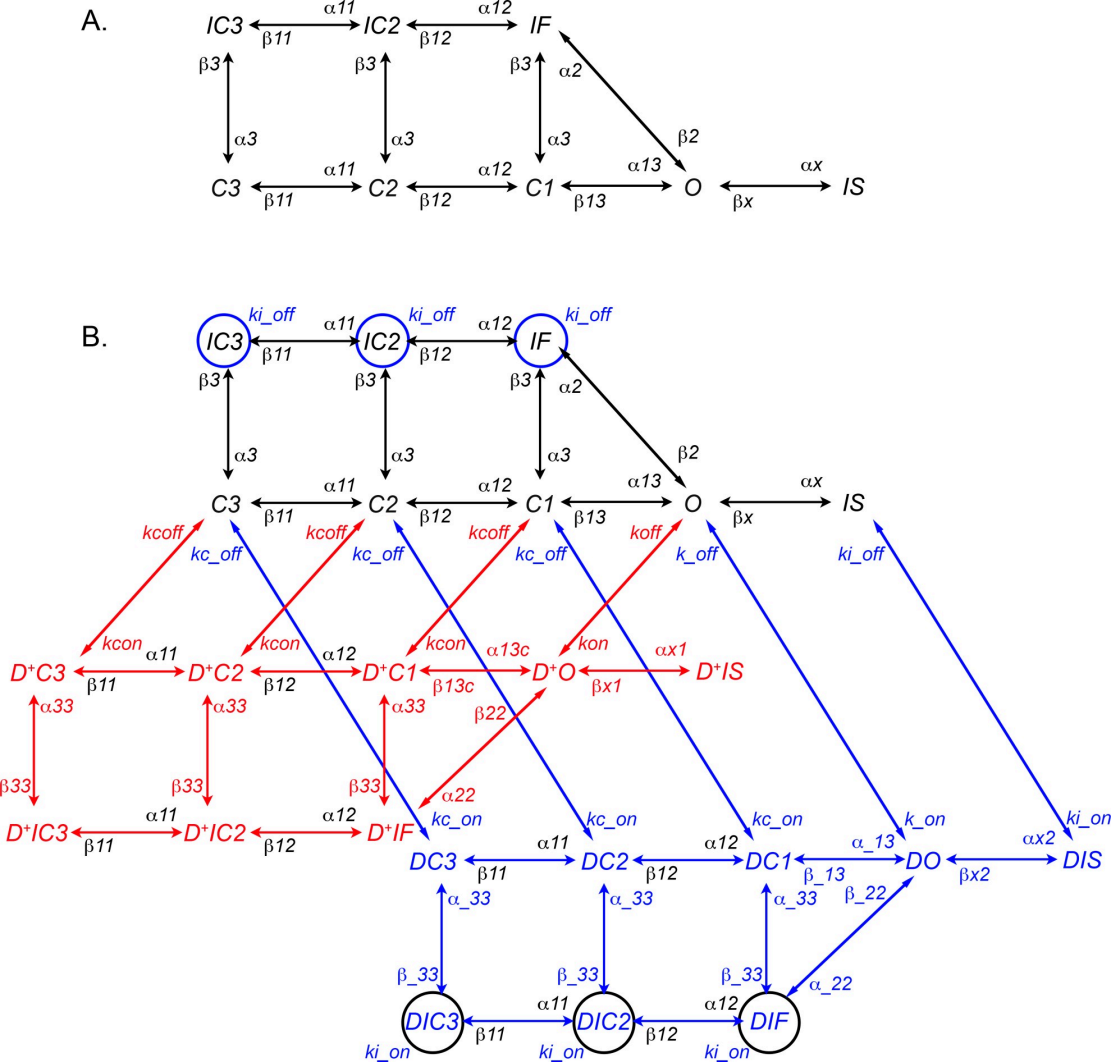
Moreno et al. [4] model of Na^+^ channel-lidocaine interactions. (A) The drug-free Na^+^ channel model. The O state represents the conducting state, while C3, C2, and C1 correspond to 3 closed states. The IC3, IC2, and IF states represent conformational states in which the “fast” inactivation gate is closed, and the IS state represents a state in which a “slow” inactivation gate is closed. Arrows indicate possible conformational state transitions with corresponding voltage-dependent rate constants labeled (e.g., α13 and β13 are the rate constants for transitions from C1 to O and O to C1, respectively). (B) The full lidocaine-Na^+^ channel interaction model. The drug-free model from (A) is depicted in black. Red (D^+^ prefix) and blue (D prefix) states represent conformational states where charged and neutral drug is bound, respectively. Charged drug can only bind to non-inactivated states (C3, C2, C1, and O), while neutral drug can bind to any state. Drug binding and unbinding rates are state-dependent, as indicated by the binding and unbinding rates of charged drug to the open state (kon and koff) and closed states (kcon and kcoff), and neutral drug to the open state (k_on and k_off), closed states (kc_on and kc_off), and inactivated states (ki_on and ki_off). For clarity, blue (black) circles as opposed to arrows were used to indicate neutral drug binding (unbinding) to the fast inactivated states with a rate constant ki_on (ki_off).

The conformational state diagram of the drug-free Moreno et al. model for Na^+^ channel dynamics is displayed in Fig 1A. Channels can be in an open state (*O*), three closed states (*C3*, *C2*, and *C1*), three fast-inactivated states (*IC3*, *IC2*, and *IF*), or a slow-inactivated state (*IS*) [4]. Conformational state transitions are indicated by arrows, with the corresponding voltage-dependent rate constants of each transition indicated by *α’*s and *β’*s.

The Moreno et al. model includes drug-channel interactions for both the neutral and charged forms of lidocaine. In Fig 1B, the interaction between charged lidocaine and Na^+^ channels is shown by the red conformational state labels and transition arrows. Lidocaine’s binding site is located in the interior of the Na^+^ channel pore, which the charged form of lidocaine can only access from the intracellular side of a non-inactivated channel [9]. Hence, charged drug can only bind to and unbind from non-inactivated conformational states (*O*, *C1*, *C2*, and *C3*). Moreno et al. adopted the modulated receptor hypothesis [14]: When charged drug is bound to the Na^+^ channel, the channel can undergo the same transitions as when no drug is bound but some transition rates are altered by drug binding.

The interaction of neutral lidocaine with Na^+^ channels is shown by blue conformational state labels and transition arrows in Fig 1B. Again, Moreno et al. use the modulated receptor hypothesis. However, unlike charged lidocaine, neutral lidocaine can access the binding site when the channel is in either inactivated or non-inactivated states [9]. Therefore, Moreno et al. allow neutral lidocaine to bind and unbind to Na^+^ channels in any conformational state, but the rate constants of drug binding are state-dependent.

Assuming mass action kinetics, the dynamics of the fraction of channels in each conformational state are given by a system of 7 differential equations for the no drug case and 23 differential equations for the full lidocaine-Na^+^ channel interaction model (see S6 Appendix for equations).

### 2.2. Analysis of the Moreno et al. model shows that lidocaine preferentially binds to and stabilizes the inactivated state of Na^+^ channels

Below, we examine the kinetics of the “detailed” Moreno et al. model and formulate a set of approximations about lidocaine’s interaction with the Na^+^ channel. We then use these assumptions to inform the structure of our low-dimensional lidocaine-Na^+^ channel interaction model in Subsection 2.3.

- *Approximation 1*: *Charged lidocaine has no effect on Na*^***+***^ *channel kinetics*.

Charged and neutral forms of lidocaine have similar concentrations at physiological pH. Using lidocaine’s pKa of 7.6 [4,9], Moreno et al. estimate that ~60% of lidocaine is positively charged and ~40% is neutral at physiological pH. On the other hand, the binding affinity of neutral lidocaine to inactivated channels is two orders of magnitude larger than that of charged lidocaine. Specifically, the dissociation constants, *K*_*d*_, of neutral lidocaine binding to non-inactivated channels is 6.8 *μM*, whereas the *K*_*d*_ for charged lidocaine binding to inactivated channels ranges from 188 to 2590 *μM* for physiological membrane potentials at 37°C [4,9–11]. Therefore, lidocaine bound to Na^+^ channels will predominantly be in the neutral form (see S1 Appendix for further details), hence we will only include the effects of the neutral form of lidocaine in our low-dimensional model of the lidocaine-Na^+^ channel interaction.

- *Approximation 2*: *Neutral lidocaine only binds to and unbinds from inactivated Na*^*+*^ *channels*.

In the Moreno et al. model, neutral lidocaine can bind to both inactivated and non-inactivated conformational states. However, the *K*_*d*_ of neutral lidocaine binding to inactivated channels (6.8 *μM*) is two orders of magnitude smaller than that for binding to non-inactivated channels (400 *μM* and 1800 *μM* for closed or open channels, respectively) [4,9,11]. Hence, we will assume that neutral lidocaine can only bind to inactivated channels in our low-dimensional model.

- *Approximation 3*: *Binding of neutral lidocaine locks Na*^*+*^ *channels in the inactivated state until the drug unbinds*.

Simulations of the Moreno et al. model show that following lidocaine binding to Na^+^ channels in the inactivated state, almost all channels remain in the inactivated state until drug unbinds (see S1 Appendix). This result arises from the transition rate constants of bound Na^+^ channels in the Moreno et al. model, which are such that when drug binds to channels, the inactivated state is substantially stabilized. This stabilization of the inactivated state can be quantified by the “relative stability” of inactivated states, which we define as the ratio of the occupancy of non-inactivated states to the occupancy of inactivated states at steady state. Over the entire physiological range of *V*, the stability ratios for drug bound channels are always less than 0.1, which implies that the binding of neutral lidocaine effectively locks the Na^+^ channel in an inactivated conformation until drug unbinds (see S1 Appendix). Therefore, in our low-dimensional model, we assume that when neutral lidocaine binds to inactivated channels, drug bound channels cannot recover from inactivation until drug unbinds.

### 2.3. A low-dimensional model for lidocaine-Na^+^ channel interaction

To construct a low-dimensional model of Na^+^ conductance, we utilize a Hodgkin-Huxley formulation for drug-free Na^+^ channel dynamics. The model has three activation gates and one inactivation gate; such that the fraction of open channels, *f*_*open*_, is *f*_*open*_ = *m*^3^*h*. The differential equations governing the dynamics of the fraction of activation and inactivation gates that are open (*m* and *h*, respectively) are

$$\frac{dm}{dt}=\alpha_{m}(1-m)-\beta_{m}m$$

$$\frac{dh}{dt}=\alpha_{h}(1-h)-\beta_{h}h,$$ (2)

where *α*_*m*_, *β*_*m*_, *α*_*h*_ and *β*_*h*_ are voltage-dependent rate constants of the form $c_{1}e^{\frac{V}{c_{2}}}$ (parameters are provided in S7 Appendix).

We extend this low-dimensional Na^+^ channel model to include the effects of lidocaine by implementing the assumptions from the preceding section. Specifically, we introduce an additional gating variable, *b*, which represents the fraction of channels bound to neutral lidocaine [15,16] (by Approximation 1, charged lidocaine plays a negligible effect). Thus, in the presence of drug, the fraction of open channels is

$$f_{open}=m^{3}h(1-b).$$

To derive the equation governing the dynamics of *b*, note that neutral lidocaine can only bind to inactivated channels (by Approximation 2), so the binding rate is modulated by the fraction of channels that are inactivated and available for binding. Thus, the instantaneous drug binding rate is *k*_*on*_[*D*](1−*h*). Then, as all drug bound channels are locked in the inactivated state (by Approximation 3), drug unbinding is not inhibited by non-inactivated channels “trapping” the drug, and the unbinding rate is simply *k*_*off*_. Therefore, the dynamics of *b* are governed by

$$\frac{db}{dt}=k_{on}[D](1-h)(1-b)-k_{off}b.$$ (3)

As in the Moreno et al. model, the drug binding rates are based on previously published values, *k*_*on*_ = 250 *M*^−1^*ms*^−1^ and *k*_*off*_ = 1.7×10^−3^ *ms*^−1^ [4,9,11]. By combining the low-dimensional Na^+^ channel model in Eq (2) with the drug binding dynamics of Eq (3), the conductance of our low-dimensional lidocaine-Na^+^ channel interaction model is $g_{Na}={\bar{g}}_{Na}m^{3}h(1-b)$.

### 2.4. Our low-dimensional model is an order of magnitude lower in dimension than the Moreno et al. model

Our low-dimensional model of the drug-free Na^+^ conductance has 2 variables and a set of *8* parameters that need to be optimized to fit experimental data, whereas the drug-free Moreno et al. model has 7 variables and 21 parameters [4]. The difference in complexity in the two models is even greater when drug-channel interactions are included in the models. The lidocaine component of our low-dimensional model consists of only 1 additional variable [15,16] and 3 additional parameters for a total of 3 variables and 11 parameters. The lidocaine component of the Moreno et al. model has 16 additional variables and 19 additional parameters for a total of 23 variables and 40 parameters. In both lidocaine interaction models, values for drug binding rates are taken from the literature [9–11], and drug concentrations, [*D*], are set by experimental protocols and pH calculations [4]. Therefore, no further parameter optimization is required to set the values of the 3 additional parameters of our low-dimensional model. On the other hand, in the Moreno et al. model, 10 further parameters describing drug-dependent state transition rates were required to be optimized to data. Altogether, our lidocaine-Na^+^ channel interaction model is an order of magnitude lower in dimension than the Moreno et al. model (3 compared to 23 variables) and has nearly four times fewer free parameters (8 compared to 31).

## 3. Results

### 3.1. Both the Moreno et al. and low-dimensional models reproduce experimental voltage-clamp data

The validity of the Moreno et al. model and our low-dimensional model are assessed by their abilities to reproduce previously published voltage-clamp data [9,10,17–20] that characterize the kinetics of the Na^+^ conductance in the presence and absence of lidocaine. In this section, we fit our low-dimensional model to the same experimental data that Moreno et al. used to fit their model and then compare the quality of fits for the two models. The fits to individual experimental voltage-clamp protocols are examined for drug-free conditions (Subsection 3.1.1) and in the presence of lidocaine (Subsection 3.1.2). We find that the low-dimensional model performs similarly well to the Moreno et al. model in recapitulating the data, despite having fewer free parameters for fitting to data.

#### 3.1.1. Drug-free models

**Fig 2:**
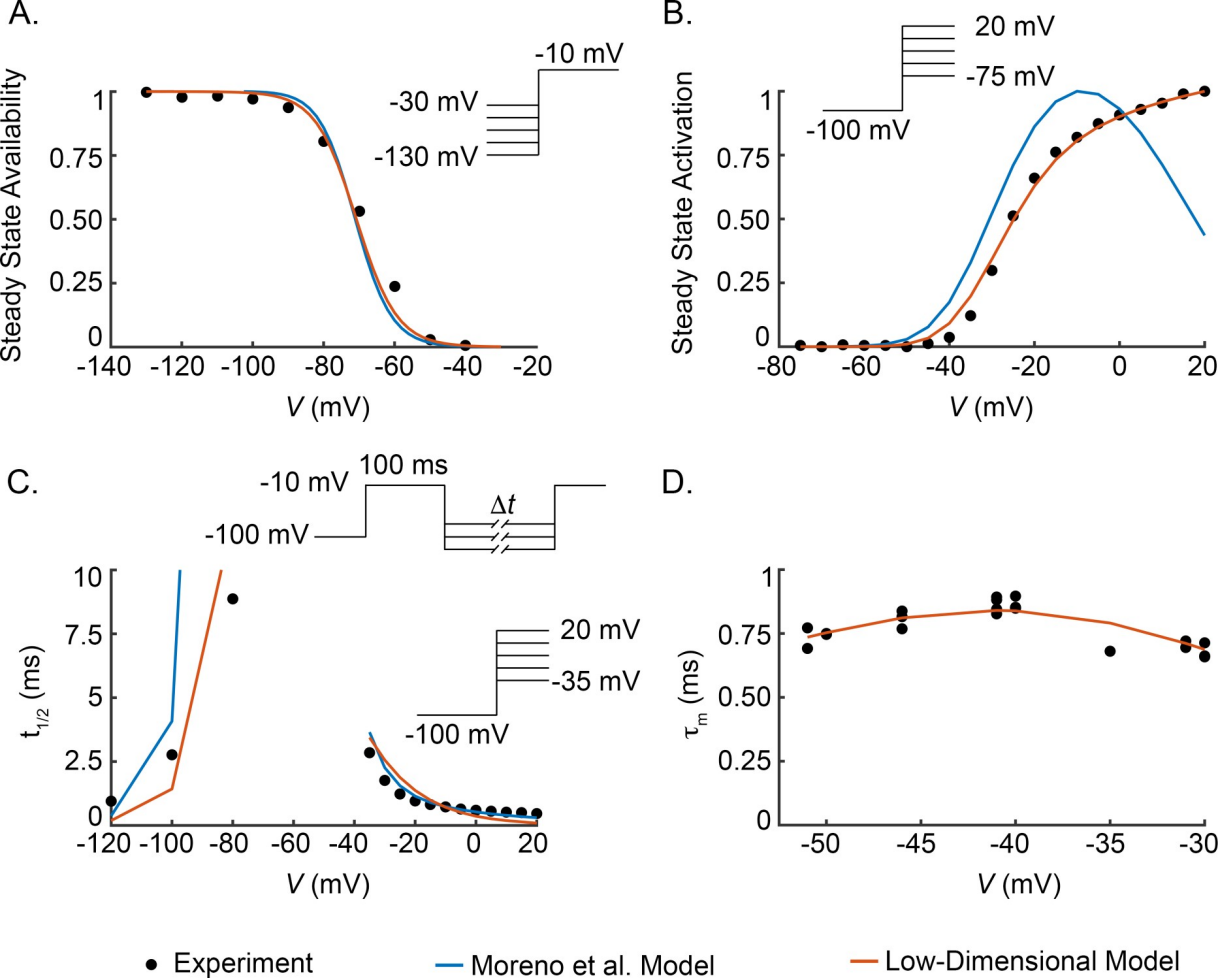
Drug-free data. The low-dimensional Na^+^ conductance model is fit to experimental data from steady state availability (A; sum squared errors (SSE) of 0.038 and 0.035 for the Moreno et al. and low-dimensional models, respectively), steady state activation (B; SSE of 0.042 and 7.0×10^−4^ for the Moreno et al. and low-dimensional models, respectively), time to half inactivation (C; SSE of 197 and 17 for the Moreno et al. and low-dimensional models, respectively), and time constant of activation (D; SSE of 1.4×10^−3^ for the low-dimensional model) voltage-clamp experiments [17–19]. In all subfigures, filled circles indicate experimental data points, blue curves represent output from the Moreno et al. model [4], orange curves represent output from the low-dimensional Na^+^ conductance model. t_1/2_ at -80 mV in the Moreno et al. model is not indicated in the figure as it is substantially larger (48.8 ms) than the other t_1/2_ values. For the low-dimensional model in (D), $\tau_{m}=\frac{1}{\alpha_{m}+\beta_{m}}.\;SSE=\frac{1}{n}\sum_{i=1}^{n}{\left(y_{i}-x_{i}\right)}^{2}$, *n* is total number of data points, {*y*_*i*_} are the model output, and {*x*_*i*_} are the experimental data values.

To appropriately compare the Moreno et al. model and our low-dimensional model of the lidocaine-Na^+^ channel interaction, the baseline Na^+^ conductance models need to exhibit similar kinetics. Therefore, we fit our drug-free Hodgkin-Huxley (*m*^3^*h*) Na^+^ conductance model, Eq (2), to voltage-clamp experimental data that Moreno et al. used to fit their model. Specifically, we fit the low-dimensional model to the steady state availability [17], steady state activation [18], and time to half inactivation data [18,20] (Fig 2A, 2B and 2C) that was used to constrain the Moreno et al. model. Note, time to half inactivation is based on two protocols; for hyperpolarized potentials, estimates were obtained from recovery from inactivation time courses, and for depolarized potentials, time required for conductance to decay 50% following an activation pulse was measured. To constrain the activation rates, the low-dimensional model is also fit to activation rate data (Fig 2D) [19]. Full experimental protocol details are provided in the S2 Appendix.

Fig 2 illustrates that, despite our low-dimensional model having only 8 free parameters and the Moreno et al. model having 21 free parameters, both models are able to replicate the experimental data. In particular, the Moreno et al. model and our low-dimensional model exhibit very similar output for steady state availability (Fig 2A) and time constant of inactivation (Fig 2C). In the steady state activation protocol (Fig 2B), our low-dimensional model outperforms the Moreno et al. model. Finally, the activation time constants of the low-dimensional model closely match experimentally determined activation rate constants (Fig 2D).

#### 3.1.2. Lidocaine-Na^+^ channel models

Having established the parameterization of the drug-free models, we now examine the ability of the lidocaine components of the models to reproduce previously published voltage-clamp data [9,10,17] that characterize the effects of lidocaine. As stated in Subsection 2.4, for both models, the drug binding and unbinding rates were set to previously published values [9–11]. We stress at the onset that no further parameters in our low-dimensional model were fit to this data, whereas 10 additional parameters were used to fit the Moreno et al. model to the data. Therefore, the results that follow show the fits of the Moreno et al. model but strictly serve only as validation for our low-dimensional drug-channel interaction model.

**Fig 3:**
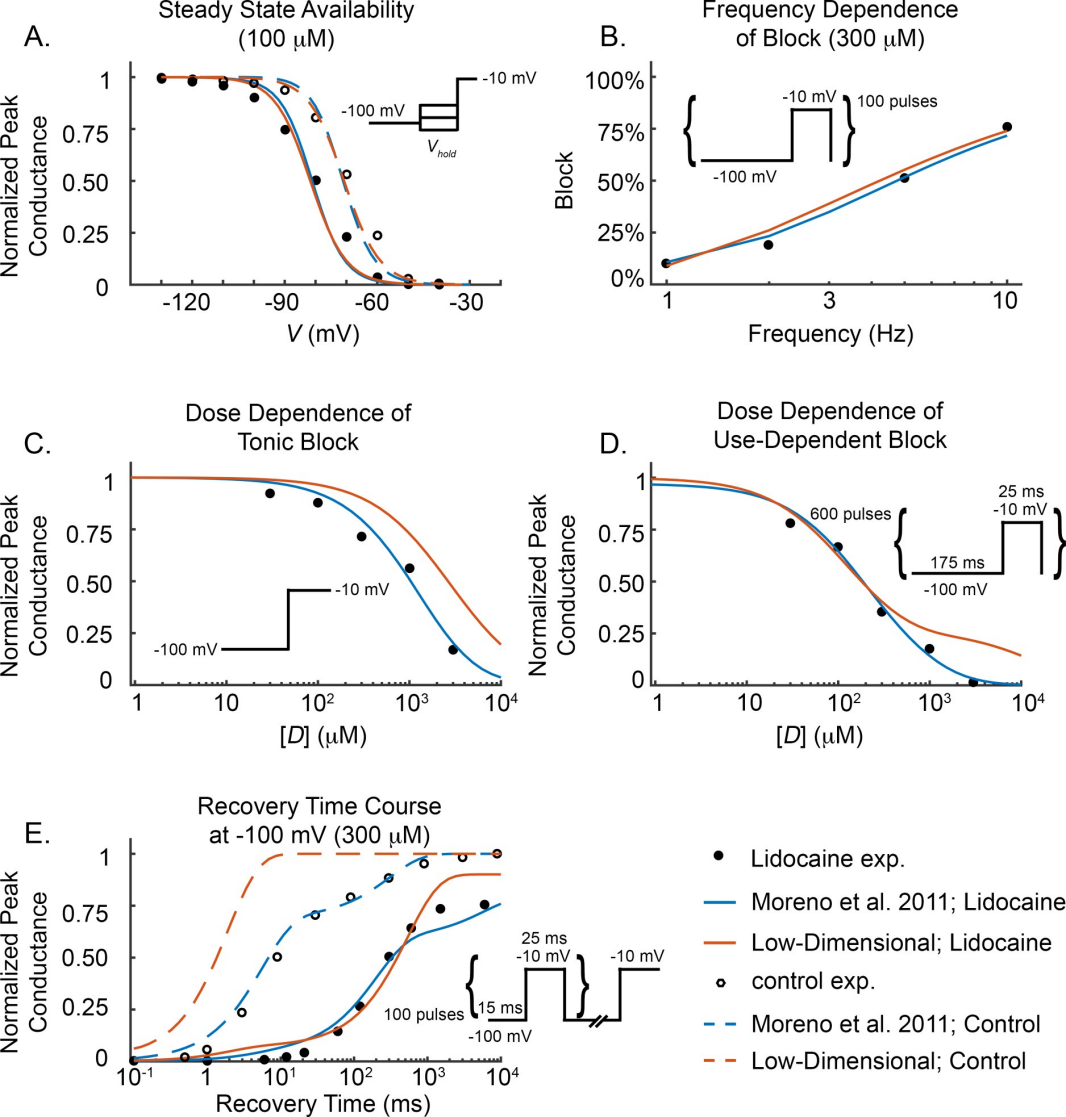
Lidocaine voltage-clamp data. Experimental voltage-clamp data (filled circles), Moreno et al. 2011 model fit (solid blue curves), low-dimensional model output (solid orange curves) for lidocaine effects on the Na^+^ conductance. *V*-clamp data for control, i.e. no drug, experiments (open circles), Moreno et al. (blue dashed curves), and low-dimensional model with no drug (orange dashed curves) are included for appropriate protocols. (A) Steady state availability with SSE of 3.6×10^−3^ and 2.9×10^−3^ for the Moreno et al. and low-dimensional lidocaine models, respectively. (B) Frequency dependence of block with SSE of 8.8×10^−4^ and 1.7×10^−3^ for the Moreno et al. and low-dimensional lidocaine models, respectively. (C) Tonic block with SSE of 3.1×10^−3^ and 0.033 for the Moreno et al. and low-dimensional lidocaine models, respectively. (D) Dose-dependence of use-dependent block with SSE of 1.3×10^−3^ and 1.1×10^−2^ for the Moreno et al. and low-dimensional lidocaine models, respectively. (E) Recovery from use-dependent block with SSE of 2.3×10^−3^ and 5.0×10^−3^ for the Moreno et al. and low-dimensional lidocaine models, respectively. $SSE=\frac{1}{n}\sum_{i=1}^{n}{\left(y_{i}-x_{i}\right)}^{2},$ *n* is total number of data points, {*y*_*i*_} are the model output, and {*x*_*i*_} are the experimental data values [9,10,17].

We consider five voltage-clamp data sets, including steady state availability, tonic block (i.e., the steady state fraction of channels bound to drug), dose-dependence of use-dependent block, recovery from use-dependent block, and frequency dependence of block (details of the experimental protocols are provided in S2 Appendix) [9,10,17]. Fig 3 displays the voltage-clamp data in the absence and presence of lidocaine (open and closed circles), and the corresponding output from the Moreno et al. model (blue curves) and our low-dimensional model (orange curves).

Steady state availability predicted by our low-dimensional model for lidocaine-Na^+^ channel interaction (Fig 3A; orange curve) agrees well with the experimental data (filled circles) [9], as does the fit of the Moreno et al. model (blue curve). The frequency dependence of block protocol (Fig 3B) [10], is also closely replicated by the low-dimensional and Moreno et al. models. Our low-dimensional model replicates the dose-dependence of tonic block and use-dependent block data (Fig 3C and 3D, respectively) [10] at the clinically relevant range of 5 to 20 *μM* [21], but slightly under predicts the fraction of blocked channels at drug concentrations much higher than the clinically relevant range. (The disparity between the low-dimensional model and tonic block data above the clinically relevant range is explained in S3 Appendix.) The Moreno et al. model performs slightly better at replicating dose-dependent block at the higher concentrations examined experimentally. Despite the recovery from inactivation of the low-dimensional model being too quick in the drug-free case (Fig 3E, dashed orange curve), the recovery from drug binding at *-*100 *mV* time course of the low-dimensional model (solid orange curve) performs similarly well to the Moreno et al. model (solid blue curve) in replicating the data.

### 3.2. Functional effects of lidocaine

The previous subsection demonstrated that both the low-dimensional model and the Moreno et al. model are able to replicate Na^+^ conductance voltage-clamp data both in the presence and absence of lidocaine. However, the primary role of mathematical models of drug-ion channel interactions is not simply to fit/predict voltage-clamp experimental results; it is to identify the mechanisms by which drugs affect dynamics at the cellular and tissue levels (e.g., peak upstroke velocity and conduction velocity), and therefore alter the propensity for arrhythmias [2,5]. First, we run simulations to systematically examine and compare the rate-dependent effects of lidocaine on peak upstroke velocity and conduction velocity in the Moreno et al. and low-dimensional models. Then, we utilize the relatively simple structure of the low-dimensional model to uncover the mechanisms underlying lidocaine’s rate-dependent effects.

#### 3.2.1. Low-dimensional model and Moreno et al. model predict similar functional effects of lidocaine on upstroke velocity and conduction velocity

In order to examine the rate-dependent effects of lidocaine on peak upstroke velocity and conduction velocity of the cardiac action potential, we incorporate both lidocaine-Na^**+**^ channel interaction models into the ten Tusscher et al. model for human ventricular myocytes [22,23] (see S4 Appendix for details of implementation of Na^+^ conductance models into the ten Tusscher et al. model). Peak upstroke velocities (and conduction velocities) in cases with drug present are normalized by the drug-free peak upstroke velocity (conduction velocity) at the same basic cycle length (BCL). (Non-normalized peak upstroke velocities and conduction velocities are provided in S4 Appendix.)

**Fig 4:**
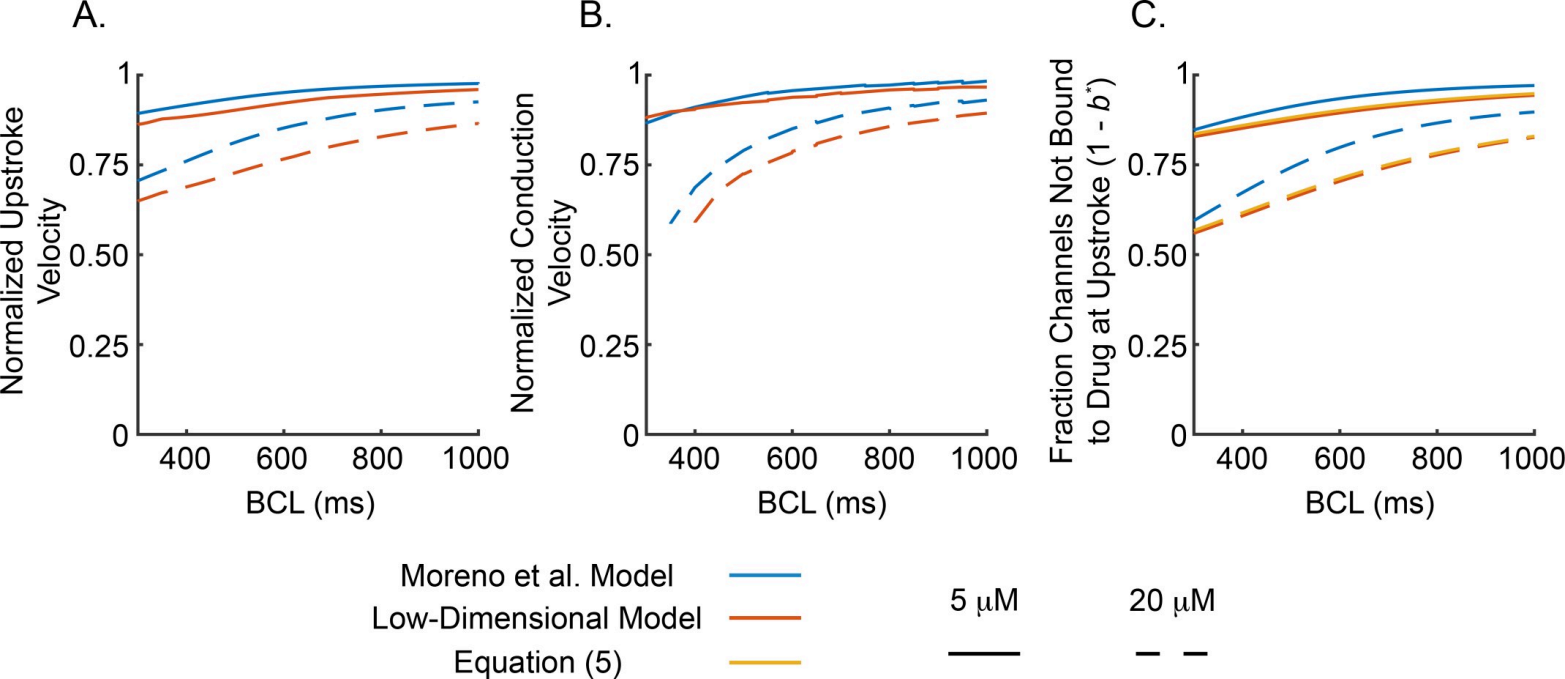
Rate-Dependent Effects of Lidocaine. Normalized peak upstroke velocity (A), conduction velocity (B), and fraction of channels not bound to drug during the upstroke (C) plotted against BCL for the ten Tusscher et al. human ventricular myocyte model [22,23] with the Moreno et al. model (blue curves) or low-dimensional model (orange curves) of the Na^+^ conductance. Peak upstroke and conduction velocities for *5 μM* (solid curves) and *20 μM* (dashed curves) concentrations of lidocaine are normalized by peak upstroke and conduction velocities at the same BCL in the corresponding drug-free model. In C, predictions from an analytically derived expression for fraction of channels bound to drug during the upstroke (b*) in our low-dimensional model are also plotted (yellow curves; see Eq (5)) and were shifted up slightly to make them visible as they overlap the output of the ten Tusscher et al. model with our low-dimensional Na^+^ conductance model.

Fig 4A shows normalized peak upstroke velocities for BCLs ranging from 300 *ms* to 1000 *ms* (i.e., the full “physiological range”) for the Moreno et al. model (blue curves) and low-dimensional model (orange curves) with drug concentrations of 5 *μM* (solid curves) and 20 *μM* (dashed curves), which correspond to low and high clinical plasma concentrations [21]. Both the low-dimensional and Moreno et al. models predict larger decreases in peak upstroke velocity due to lidocaine block as BCL decreases. At a drug concentration of 20 *μM*, both models predict approximately a 10% reduction in peak upstroke velocity at a BCL of 1000 *ms* (7.5% and 13.5% reductions in the Moreno et al. and low-dimensional models, respectively), whereas upstroke velocity is decreased by approximately 30% at BCL of 300 *ms* (29% and 35% reductions in the Moreno et al. and low-dimensional models, respectively).

Fig 4B displays normalized conduction velocities for lidocaine concentrations of 5 *μM* (solid curves) or 20 *μM* (dashed curves) as a function of BCL. Note that the curves for 20 *μM* lidocaine terminate at BCLs of 350 and 400 *ms* for the Moreno et al. (blue curves) and low-dimensional (orange) models, respectively, because pacing with shorter BCLs did not elicit propagating waves for each stimulus. As with upstroke velocity, both models predict more prominent effects of lidocaine block as BCL decreases, i.e., there is a greater reduction in conduction velocity at lower BCL. Specifically, at a lidocaine concentration of 20 *μM*, both models predict approximately a 10% reduction in normalized conduction velocity for a BCL of 1000 *ms* (7% and 10% for the Moreno et al. and low-dimensional models, respectively), and this reduction increases to approximately 35% as BCL decreases to 400 *ms* (31% and 40% for the Moreno et al. and low-dimensional models, respectively). We also examine the effect of lidocaine on conduction velocity restitution following a premature beat as opposed to during steady pacing (Fig 4B) and find conduction velocity restitution is similar for premature beats and constant pacing in both the Moreno et al. and our low-dimensional model (Figs 2 and 3 in S4 Appendix).

The available Na^+^ conductance during the upstroke, and hence the fraction of channels bound to drug during the upstroke (b*), is considered to be one of the primary determinants of peak upstroke velocity and conduction velocity [2]. The fraction of channels not bound to drug during the upstroke (1−b*) as a function of BCL in the ten Tusscher et al. model with either the Moreno et al. (blue curves) or our low-dimensional (orange curves) Na^+^ conductance models are plotted in Fig 4C. 1−b* has the same rate-dependence as normalized peak upstroke and conduction velocity, verifying the strong causal link between these quantities and the level of block of Na^+^ channels by lidocaine.

#### 3.2.2. Rate-dependent effects of lidocaine arise from voltage-dependence of Na^+^ channel inactivation: Insight from analysis of low-dimensional model

In this section, we analyze our low-dimensional model, and by capitalizing on its relative simplicity, we elucidate the mechanisms underlying the rate-dependent effects of lidocaine on upstroke velocity and conduction velocity. Specifically, we derive an expression for the fraction of Na^+^ channels bound to drug during the upstroke (b*), similar to that derived by Starmer et al. [24] and Weirich and Antoni [25]. This expression captures the parametric dependence on BCL, action potential duration restitution properties, diastolic potential, channel inactivation kinetics, and drug concentration and binding rates.

**Fig 5:**
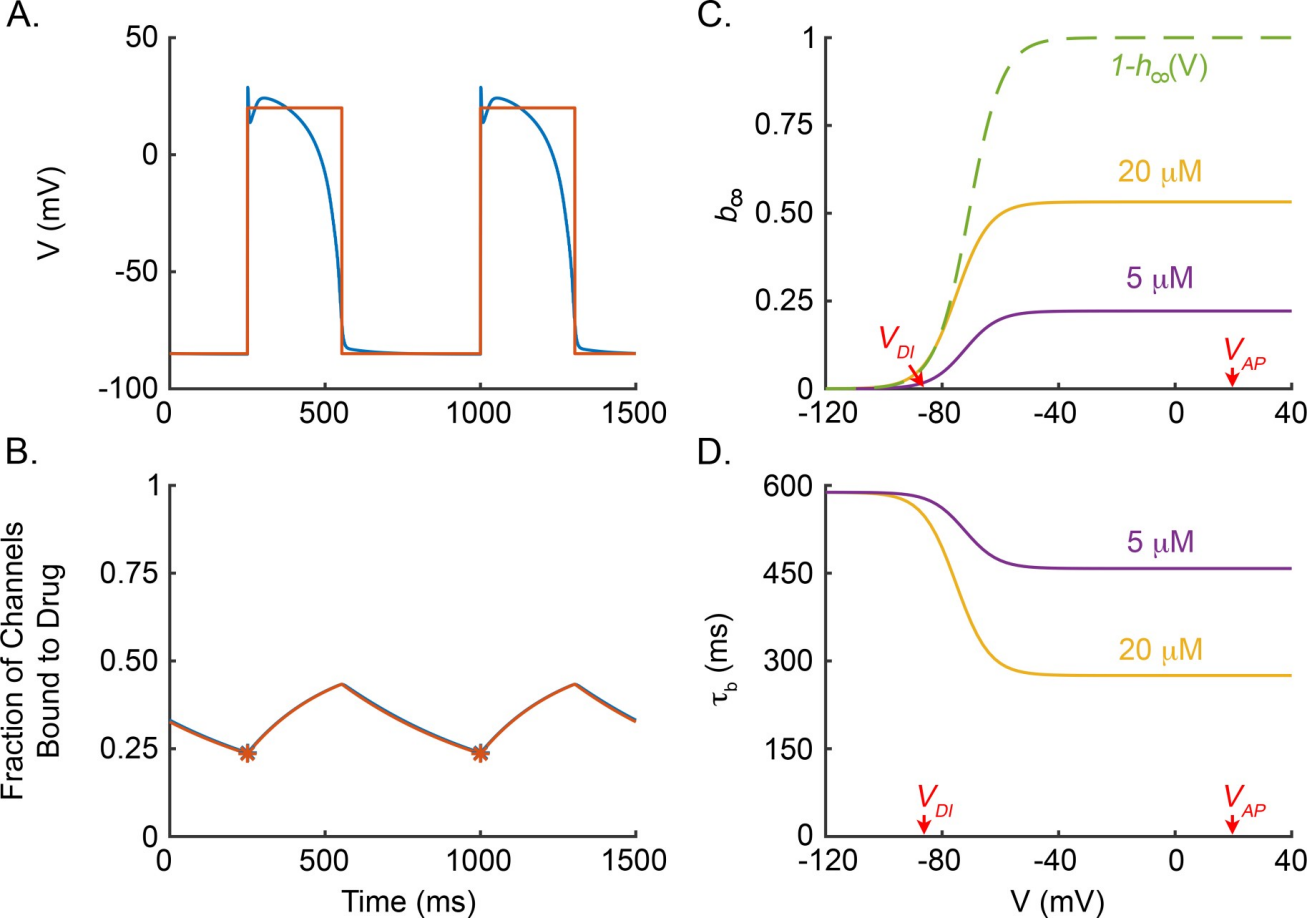
Mechanism of lidocaine rate-dependent effects. (A) Simulated action potentials of our modified ten Tusscher et al. model paced at a BCL of 750 ms with *20 μM* lidocaine (blue) and a square wave approximation of the action potential (orange) that alternates between *-85 mV* and *20 mV*. (B) Dynamics of the fraction of channels bound to drug, *b*, with *20 μM* lidocaine present in our modified ten Tusscher et al. model (blue) and low-dimensional Na^+^ conductance model stimulated by the square wave approximation (orange). (C) Voltage dependence of 1−*h*_∞_ (dashed green) and b_∞_ for *20 μM* (yellow) and *5 μM* (purple) lidocaine. (D) Voltage dependence of *τ*_*b*_ for *20 μM* (yellow) and *5 μM* (purple) lidocaine.

**Fig 6:**
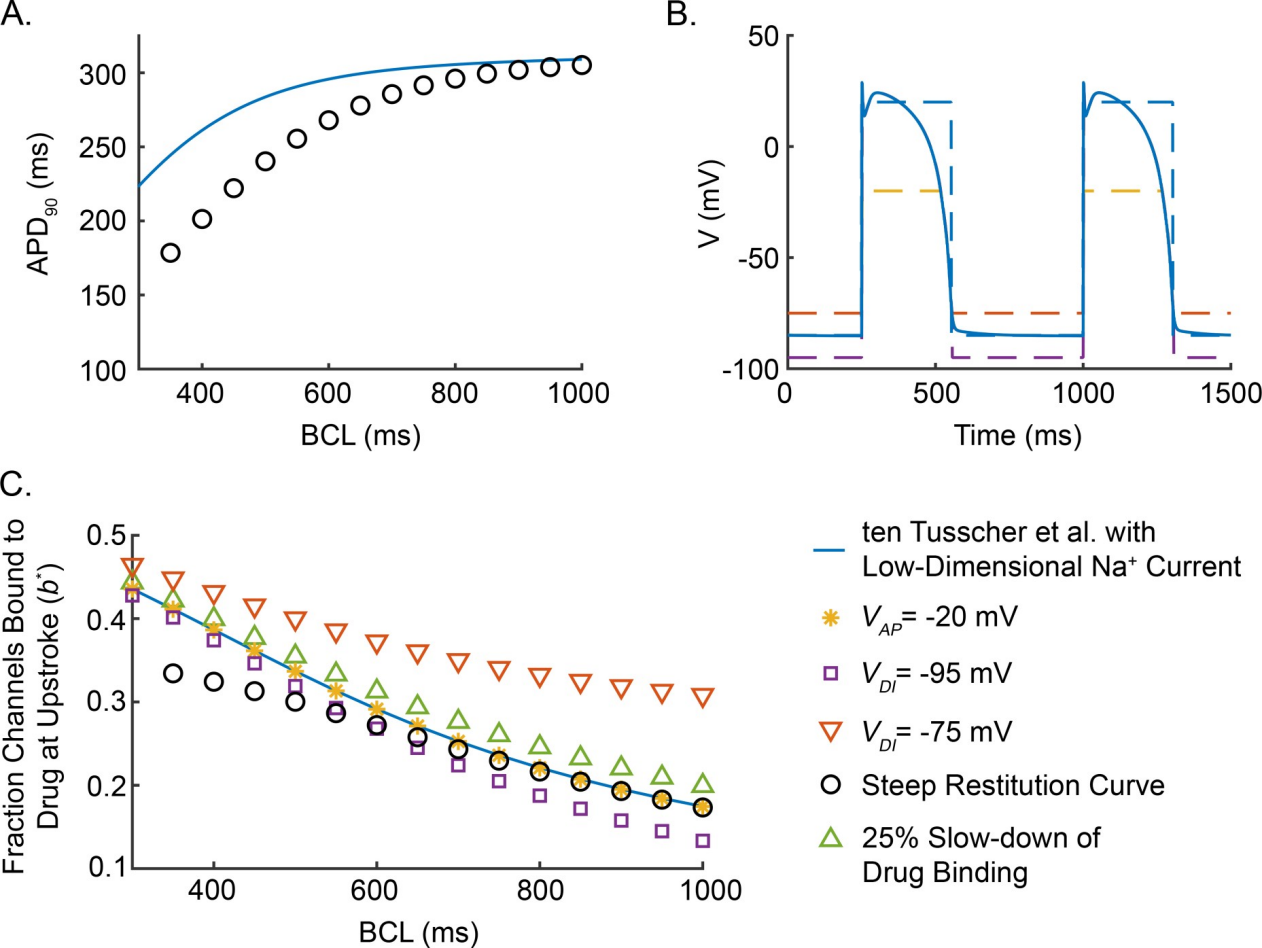
Cardiac electrophysiological properties and lidocaine binding. (A) APD_90_ restitution curve for our modified ten Tusscher et al. model with *20 μM* of lidocaine (blue curve) and a steeper, hypothetical restitution curve (black circles). (B) Time course of transmembrane potential for our modified ten Tusscher et al. model paced at a BCL of *750 ms* and the square wave approximation of *V* (dashed blue line) as well as hypothetical alterations: *V*_*AP*_ decreased to *-20 mV* (dashed yellow curve), *V*_*DI*_ decreased to *-95 mV* (dashed purple curve), and *V*_*DI*_ increased to *-75 mV* (dashed orange curve). (C) Fraction of channels bound to lidocaine during the upstroke (b*(BCL)) in the presence of *20 μM* lidocaine, as given by Eq *(5)*, for our modified ten Tusscher et al. model and the various shifts in cardiac electrophysiological characteristics in (A) and (B).

We first note that, when the ten Tusscher et al. model with our low-dimensional Na^+^ conductance model (from now on referred to as “our modified ten Tusscher et al. model”) is paced at a constant BCL, the membrane potential (*V*) is approximately -85 *mV* during the diastolic interval (DI), and *V* is approximately 20 *mV* for the duration of the action potential (APD) (Fig 5A; blue curve). Therefore, we approximate the time course of *V* by a square wave alternating between -85 *mV* and 20 *mV* (Fig 5A; orange curve). Furthermore, throughout the DI and AP, the fraction of non-inactivated Na^+^ channels, *h*, is approximately at the steady state values of *h*_∞_(−85 *mV*)≈0.9 and *h*_∞_(20 *mV*)≈0, respectively. Consequently, we approximate the time course of *h* as a square wave alternating between *h*_∞_(−85 *mV*) and *h*_∞_(20 *mV*). For a given BCL, we take the APD and DI of our square wave approximations to be given by the APD_90_ restitution curve of our modified ten Tusscher et al. model (i.e., APD is a function of BCL as displayed by the blue curve in Fig 6A, and *DI* = *BCL*−*APD*), noting that the effect of lidocaine on the restitution curve is negligible other than shifting the minimum BCL at which an action potential is induced following each stimulus.

By exploiting the piecewise constant nature of the square wave approximations of *V* and *h*, we are able to solve Eq (3) to obtain formulae describing the fluctuations of the fraction of Na^+^ channels bound to drug for arbitrary BCL,

$$b(t)=\left\{ \begin{array}{c} b_{\infty }(V_{AP})-(b_{\infty }(V_{AP})-b(t_{k}))e^{-\frac{t-t_{k}}{\tau_{b}(V_{AP})}},\;t_{k}\leq t<t_{k}+APD\\ b_{\infty }(V_{DI})-(b_{\infty }(V_{DI})-b(t_{k}+APD))e^{-\frac{t-(t_{k}+APD)}{\tau_{b}(V_{DI})}},\;t_{k}+APD\leq t<t_{k+1} \end{array}\right.,$$ (4)

where *t*_*k*_ is the time of the k^th^ upstroke, *b*(*t*_*k*_) is the fraction of channels bound to lidocaine at the time of the upstroke, and

$$\tau_{b}(V)=\frac{1}{(1-h_{\infty }(V))[D]k_{on}+k_{off}},b_{\infty }(V)=\frac{(1-h_{\infty }(V))[D]k_{on}}{(1-h_{\infty }(V))[D]k_{on}+k_{off}}.$$

Fig 5B shows that the dynamics of the fraction of channels bound to drug, *b*(*t*), given by the approximation in Eq (4) and direct simulations of our modified ten Tusscher et al. model (orange and blue curves, respectively) are in such close agreement that the curves are nearly indistinguishable.

The steady state fraction of channels bound to drug during the upstroke (b*) can be found by setting the fraction of channels bound to drug during the upstroke at time *t*_*k*_ equal to the fraction of channels bound during the subsequent upstroke at time *t*_*k+1*_,

$$b(t_{k})=b(t_{k+1})=b^{*}.$$

Solving this equation yields

$$b^{*}=\left[\frac{1-D}{1-AD}\right]b_{\infty }(V_{DI})+\left[\frac{(1-A)D}{1-AD}\right]b_{\infty }(V_{AP}),$$ (5)

where $A=e^{-\frac{APD}{\tau_{b}(V_{AP})}}$ and $D=e^{-\frac{DI}{\tau_{b}(V_{DI})}}$. Fig 4C plots the rate-dependence of 1−b* as predicted by the algebraic expression in Eq (5) (yellow curves) along with the results from direct simulations of our modified ten Tusscher et al. model (orange curves). These curves are indistinguishable on the scale of the figure, indicating that Eq (5) provides an excellent approximation for the level of drug binding during periodic pacing.

Eq (5) implies that, under constant pacing, the fraction of channels bound to drug during the upstroke (b*) is a weighted sum of the steady state fraction of channels bound to drug at the plateau potential (*b*_∞_(*V*_*AP*_)) and the steady state fraction of channels bound to drug at the diastolic potential (*b*_∞_(*V*_*DI*_)) (Fig 5C). The weight factors are dependent on BCL through APD and DI as dictated by the restitution curve (blue curve in Fig 6A), as well as the effective time constants of drug binding at the plateau potential (*τ*_*b*_(*V*_*AP*_)) and the diastolic potential (*τ*_*b*_(*V*_*DI*_)) (Fig 5D). Thus, Eq (5) provides an efficient way to examine the dependence of drug binding on heart rate and cellular dynamics (i.e., through restitution properties, and diastolic and plateau potentials), as well as drug concentration and binding kinetics. Furthermore, analysis of Eq (5) can help identify the mechanisms underlying the rate-dependencies.

- *Steeper restitution curve decreases rate-dependence of lidocaine binding*.

Fig 6A displays the restitution curve of our modified ten Tusscher et al. model (blue curve) and a hypothetical, steeper restitution (black circles), which we use to illustrate the effect of restitution properties on the rate-dependence of lidocaine block. Fig 6C plots the corresponding rate-dependence of the fraction of Na^+^ channels blocked by drug during the upstroke of the action potential (b*) as predicted by Eq (5) in the presence of 20 *μM* of lidocaine. At high BCL, b* increases as BCL decreases for both the default and the steeper restitution curves. For lower BCL, where APDs in the steeper restitution curve are substantially shorter than in our modified ten Tusscher et al. model, b* continues to increase as BCL decreases, but the steeper restitution curve yields a lower level of block (b*) and a lower degree of rate-dependence than the default case.

The lower level of block in the case of the steeper restitution curve is easily understood. Note that the voltage dependence of *b*_∞_(*V*), which is inherited from *h*_∞_(*V*), indicates that lidocaine tends to bind during the AP and unbind during the diastolic interval (Fig 5C). The steeper restitution curve is associated with a smaller APD and a correspondingly larger DI for a given BCL. Therefore, there is less time for drug binding during the action potential and more time for unbinding during the DI, leading to an overall decrease in the level of bound drug. This effect is embedded in Eq (5) as a decreased weight of *b*_∞_(*V*_*AP*_) and increased weight of *b*_∞_(*V*_*DI*_) through their dependence on APD and DI.

The increased level of block (b*) with decreased BCL at high BCL can be explained in a similar manner. The restitution curve is flat for large BCL, and therefore APD remains roughly constant while DI accounts for any change in BCL. This implies that, as BCL decreases, there is less time for drug unbinding during the DI, which leads to an overall increase in the level of bound drug.

It is not immediately clear how the level of block (b*) should change with decreases in BCL at lower BCL (i.e., the portion of the restitution curves where both DI and APD change substantially with BCL). However, insight can be obtained from the sensitivity of b* with respect to BCL according to Eq (5),

$$\frac{\partial b^{*}}{\partial BCL}=\frac{b_{\infty }(V_{AP})-b_{\infty }(V_{DI})}{{\left(1-AD\right)}^{2}}\left(\frac{(1-D)AD}{\tau_{b}(V_{AP})}\frac{df(BCL)}{dBCL}-\frac{(1-A)D}{\tau_{b}(V_{DI})}(1-\frac{df(BCL)}{dBCL})\right),$$ (6)

where *APD* = *f*(*BCL*) is the restitution curve (see S5 Appendix for derivation). First note that when the restitution curve is flat (i.e., $\frac{df(BCL)}{dBCL}\approx 0$), Eq (6) confirms that b* increases with decreasing BCL (as described above). However, as the restitution curve steepens, (i.e., $\frac{df(BCL)}{dBCL}>0$), Eq (6) indicates that degree of rate-dependence will decrease. In fact, Eq (6) suggests that reverse rate-dependence is possible if

$$\frac{(1-D)AD}{\tau_{b}(V_{AP})}\frac{df(BCL)}{dBCL}>\frac{(1-A)D}{\tau_{b}(V_{DI})}\left(1-\frac{df(BCL)}{dBCL}\right).$$

In other words, there is a critical slope of the restitution curve above which reverse rate-dependent drug binding occurs,

$$\frac{df(BCL)}{dBCL}={\left(1+\frac{\tau_{b}(V_{DI})}{\tau_{b}(V_{AP})}\frac{(1-D)A}{\left(1-A\right)}\right)}^{-1}.$$

For the drug binding rates of lidocaine, we find that reverse rate-dependence would only occur for non-physiological restitution curves. However, reverse rate-dependence could occur under physiological conditions for state-dependent Na^+^ channel blockers with similar binding pathways but slower unbinding rates.

- *Lidocaine binding is insensitive to changes in AP amplitude and plateau potential*.

To examine the effect of the AP plateau potential (*V*_*AP*_) on the rate-dependent binding of lidocaine, we shift *V*_*AP*_ from 20 *mV* in our default model (Fig 6B; dashed blue curve) to a hypothetical value of *-*20 *mV* (dashed yellow curve). Fig 6C shows that the fraction of channels bound to drug during the upstroke (b*) for *V*_*AP*_ = −20 *mV* (Fig 6C; yellow asterisks) overlays the values for *V*_*AP*_ = 20 *mV* (blue curve).

Eq (5) reveals that the insensitivity of b* on *V*_*AP*_ is inherited entirely through its dependence on *b*_∞_(*V*_*AP*_) and *τ*_*b*_(*V*_*AP*_), which in turn inherit their dependence on *V*_*AP*_ through the steady state inactivation curve 1−*h*_∞_(*V*_*AP*_). (The quantitative details of the local insensitivity of b* on *V*_*AP*_ are given by the derivative $\frac{\partial b^{*}}{\partial V_{AP}}$; see S5 Appendix.) The dashed green curve in Fig 5C demonstrates that steady state inactivation is saturated at potentials above -55 *mV*, and thus, b* is insensitive to variations in *V*_*AP*_. In fact, the insensitivity of b* to variations in *V*_*AP*_ implies that, while lidocaine block in our modified ten Tusscher et al. model depends on the duration of the AP, it does not depend on the detailed shape of the AP.

- *Increases in the diastolic potential promote lidocaine binding*, *yet binding is insensitive to decreases in diastolic potential*.

The effects of decreasing and increasing the diastolic potential *V*_*DI*_ by 10 *mV* on the fraction channels bound to drug during the upstroke (b*) are illustrated in Fig 6C (purple squares and orange inverted triangles, respectively). Decreasing *V*_*DI*_ decreases b* marginally at low BCL and moderately at high BCL. On the other hand, increasing *V*_*DI*_ can substantially increase b*, especially at longer BCL. As was the case for *V*_*AP*_, the sensitivity of b* to *V*_*DI*_ is inherited from the steady state inactivation curve (Fig 5C). In our modified ten Tusscher et al. model, *V*_*DI*_≈−85 *mV*, which is just below the threshold of the steady state inactivation curve. Below *V*_*DI*_ = −85 *mV*, the steady state inactivation curve is flat with 1−*h*_∞_(*V*)≈0, causing b* to be insensitive to decreases in *V*_*DI*_; whereas above *V*_*DI*_ = −85 *mV*, 1−*h*_∞_(*V*) steepens drastically, leading b* to be highly sensitive to increases in *V*_*DI*_. (The quantitative details of the local sensitivity of b* on *V*_*DI*_ are given by the derivative $\frac{\partial b^{*}}{\partial V_{DI}}$; see S5 Appendix.)

- *Lidocaine binding is insensitive to minor changes in drug binding rate*.

Fig 6C also displays the predicted effect that decreasing lidocaine binding and unbinding rates by 25% would have on the fraction of channels bound to lidocaine during the upstroke (green triangles). The predictions for b* are only slightly higher than those obtained when we used the published estimates of *k*_*on*_ and *k*_*off*_ [4,9,11], indicating that b* is locally insensitive to changes in the time constant of lidocaine binding kinetics. Note that Eq (5) indicates that scaling *k*_*on*_ and *k*_*off*_ by a factor of *σ* is equivalent to scaling APD and DI by *σ*, and thus its effect is the same as rescaling BCL with an adjusted restitution curve, such that

$$APD=\sigma^{-1}f(\sigma \;BCL)$$

(e.g., decreasing lidocaine binding and unbinding rates by 25% is equivalent to decreasing APD and BCL by 25%).

## 4. Discussion

In this study, we construct and analyze a novel low-dimensional model for lidocaine-Na^+^ channel interaction. The structure of our model is based on (1) the mathematical framework proposed by Starmer et al. [15,16,24] for modeling state-dependent drug binding and (2) the key features of lidocaine-Na^+^ channel interactions that we identify through an analysis of a high-dimensional Markov model by Moreno et al [4]. Our low-dimensional model consists of a two-variable Hodgkin-Huxley-type Na^+^ conductance model and an additional variable for the fraction of channels bound to drug. One limitation of our model is that, to limit dimensionality, our model does not include a slow inactivation process, and as a result, our low-dimensional model recovers from inactivation faster than is seen experimentally (Fig 3E; Control). Future studies could attempt to add a slow inactivation process to more accurately reproduce recovery from inactivation. However, other than recovery from inactivation, our model fits data from an extensive set of voltage-clamp experiments to a similar degree as the Moreno et al. model, despite our model’s low-dimensionality. Furthermore, similar effects of lidocaine on action potential upstroke velocity and conduction velocity are predicted when either our model or the Moreno et al. model is incorporated into the ten Tusscher et al. model for human ventricular cells.

The results from previous voltage-clamp experiments and computer simulation studies have suggested that lidocaine preferentially binds to and stabilizes Na^+^ channels in the inactive state [11,26–28], while other studies have suggested that lidocaine binds to Na^+^ channels in other conformational states as well [9,11]. Indeed, the Moreno et al. model allows lidocaine to bind to Na^+^ channels in any state. However, our analysis of the Moreno et al. model demonstrates that the primary interaction of lidocaine with the Na^+^ channel is stabilization of the inactivation gate. Specifically, our analysis of the state transition and binding rate constants of the Moreno et al. model identifies that (1) The vast majority of lidocaine bound to Na^+^ channels is the neutral form of lidocaine; (2) Neutral lidocaine binds and unbinds almost exclusively to inactivated Na^+^ channels; and (3) Upon binding to Na^+^ channels, lidocaine effectively immobilizes the inactivation gate, such that channels cannot recover from inactivation before lidocaine unbinds. Our low-dimensional model is derived based on these three key properties of lidocaine-Na^+^ channel interactions. The close agreement between extensive voltage-clamp data and our low-dimensional model supports the hypothesis that lidocaine binding to non-inactivated channels has a negligible contribution to lidocaine’s overall effects. While the effects of charged lidocaine and binding to non-inactivated channels could be included in our model, it would come at the expense of higher dimensionality [29] and would likely only marginally improve the fits to the voltage-clamp data.

By exploiting the low-dimensionality of our model, we derive an algebraic expression for the fraction of channels bound to drug during periodic pacing (b*). Previously, Starmer et al. [24] and Weirich and Antoni [25,30] proposed similar expressions. We extend this previous work in two ways. First, we validate the key assumption that the drug binding rates during the action potentials and diastolic intervals are well approximated as constant values. Second, we use the algebraic expression for b* to explicitly explore the dependence of rate-dependent block on electrophysiological properties of cardiac cells (i.e., APD, DI, *V*_*AP*_, and *V*_*DI*_). In particular, we show that, while the level of lidocaine binding is highly dependent on action potential duration, lidocaine binding is unaffected by changes in the shape of the AP waveform (e.g., changes in plateau potential). The insensitivity of drug binding to the AP waveform results from steady state inactivation being saturated above -55 *mV*, which leads to an approximately constant binding rate of lidocaine for membrane potentials above -55 *mV*. On the other hand, because steady state inactivation, and hence lidocaine binding rate, shifts rapidly between -85 *mV* and -75 *mV*, the level of lidocaine binding is highly sensitive to increases in diastolic potential (*V*_*DI*_).

To explore the functional effect of lidocaine on action potential upstroke velocity and conduction velocity, we incorporate our low dimensional model and the Moreno et al. model for drug-channel interactions into the ten Tusscher et al. model of human ventricular cells. However, it is important to note that quantitative predictions can be highly dependent on the details of the electrophysiological models utilized [31–33]. The algebraic expression for the fraction of channels bound to drug during the action potential upstroke provides a model-independent method for assessing the influence of electrophysiological properties of cardiac cells (i.e., APD, DI, *V*_*AP*_, and *V*_*DI*_) on lidocaine binding. Hence, our qualitative observations on how lidocaine binding is affected by changes in the AP waveform or restitution properties are robust to variation in the details of the electrophysiological models.

Lidocaine is considered to have a strong cardiac safety profile [5]. While we characterize the mechanisms underlying the rate-dependent block of lidocaine in the present study, the hallmarks of a safe Na^+^ channel blocker cannot be determined from studying lidocaine alone. Rather, it requires a detailed comparison of the mechanisms underlying lidocaine’s rate-dependent effects to those of less safe Na^+^ channel blockers, such as flecainide [3]. To this end, low-dimensional models for Na^+^ channel blockers with low safety profiles that include only primary binding pathways would be exceptionally beneficial.

Our development of a low-dimensional model for lidocaine’s interaction with the Na^+^ channel benefited from two key features, and the methodology could readily be applied to other drug-ion channel interactions that have similar features. First, lidocaine has a single predominant binding site/pathway (i.e., binding to inactivated channels), and second, lidocaine stabilizes the conformational states to which it binds (i.e., inactivated states), causing the unbinding rate to be proportional to the fraction of channels bound (*k*_*off*_*b*). Together these features allow drug binding to be incorporated into the Na^+^ channel model with a single equation. Note that the equation would be just as simple if lidocaine binding had no effect on the conformational state stability of the Na^+^ channel (i.e., as in the guarded receptor model [15]). In this case, the unbinding rate would be proportional to both the fraction of channels bound to drug and the fraction of channels with the binding site open for drug egress, e.g., for inactivated state binding, the unbinding rate would be *k*_*off*_(1−*h*)*b*. On the other hand, if more than one binding site/pathway were relevant or if drug binding altered transition kinetics, model reduction could still be possible, but two or more equations would be required to describe drug-Na^+^ channel interactions.

Regardless of the methodology used to develop low-dimensional models for other Na^+^ channel blockers with low safety profiles, the knowledge gained through analysis and comparison of such models to our low-dimensional lidocaine-Na^+^ channel model could be invaluable in understanding the drug properties that characterize strong or weak safety profiles and thus greatly enhance our ability to develop new antiarrhythmic Na^+^ channel blockers that are both safe and effective.

## Supporting information

S1 Appendix Justification of lidocaine-Na^+^ channel interaction approximations. (DOCX)

S2 Appendix Experimental voltage-clamp protocols. (DOCX)

S3 Appendix Low-dimensional model under predicts tonic block at -100 *mV*. (DOCX)

S4 Appendix Peak upstroke and conduction velocity in modified ten Tusscher et al. models. (DOCX)

S5 Appendix Dependence of lidocaine binding on physiological properties. (DOCX)

S6 Appendix Moreno et al. model. (DOCX)

S7 Appendix Equations of the low-dimensional lidocaine-Na^+^ channel interaction model. (DOCX)

S8 Appendix Numerical Methods. (DOCX)
