# Supplementary material for: Rate-dependent effects of lidocaine on cardiac dynamics: Development and analysis of a low-dimensional drug-channel interaction model
Source: PLoS Comput Biol. 2021 Jun 29;17(6):e1009145. doi: 10.1371/journal.pcbi.1009145 (PMC8274935; doi:10.1371/journal.pcbi.1009145)
Supplement: S1 Appendix — (DOCX) [file pcbi.1009145.s001.docx]

# **Justification of lidocaine-Na^+^ channel interaction approximations.**

In formulating our low-dimensional model of the lidocaine-Na^+^ channel interaction (Section 2.2 of the main text), we assume that: (1) Charged lidocaine has no effect on Na^+^ channel kinetics; (2) Neutral lidocaine only binds to and unbinds from inactivated Na^+^ channels; and (3) Binding of neutral lidocaine locks Na^+^ channels in the inactivated state until the drug unbinds. Here, we augment the arguments laid out in Section 2.2 of the main text, providing further justification for our modeling assumptions.

## Direct simulations of the modified ten Tusscher et al. model demonstrate that lidocaine’s effects are due to the neutral form binding to inactivated channels

The above assumptions assert that lidocaine binding only consists of neutral drug binding to inactivated channels. In the main text, we base our assumptions on the rate constants of the Moreno et al. model [1]. To directly assess the validity of these assumptions, we simulated the ten Tusscher et al. model [2,3] with the Moreno et al. model at a BCL of $750 ms$ and recorded the fraction of Na^+^ channels bound to lidocaine and the state of the blocked channels. Fig 1 displays resulting time courses of transmembrane potential (A and C; $5 \mu M$ and $20 \mu M$ of lidocaine, respectively) and fraction of channels in inactivated states bound to neutral lidocaine, in non-inactivated states bound to neutral lidocaine, and bound to charged lidocaine (orange, yellow, and purple lines, respectively in panels B and D). At all times, the vast majority of drug bound channels are bound to the neutral form of lidocaine and are in the inactivated state (orange lines). In fact, throughout the $750 ms$ BCL displayed in Fig 1, the fraction of channels bound to neutral drug in a non-inactivated state or bound to charged drug in any state never exceed $3.8\times{10}^{-3}$ and $4.8\times{10}^{-3}$, respectively, when $\left[ D \right]=20 \mu M$. Moreover, the proportion of drug bound channels that are in an inactivated state and bound to neutral drug never falls below $95\%$ of all drug bound channels for either $\left[ D \right]=5 \mu M$ or $20 \mu M$.


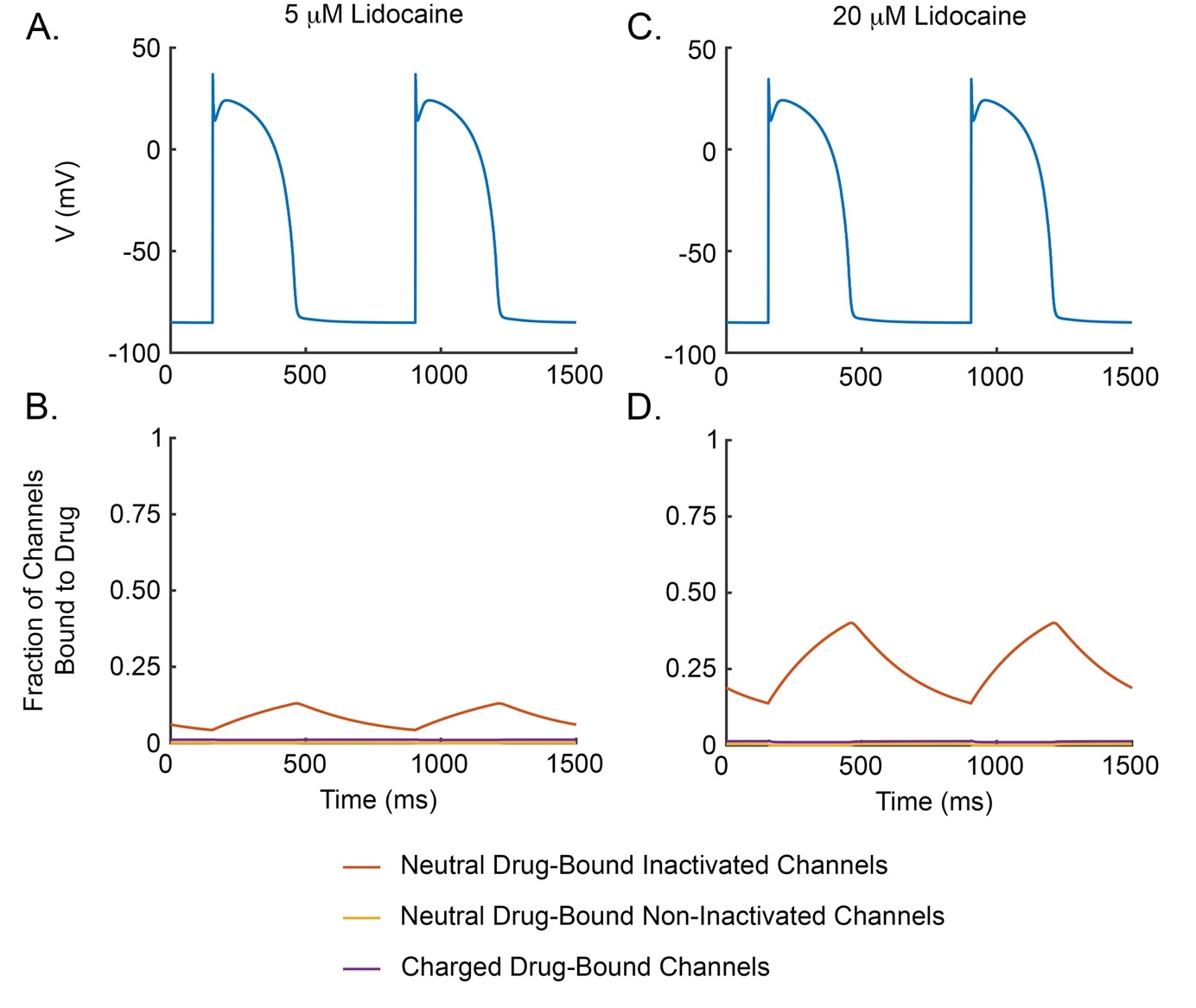


**Fig 1: Fraction of channels bound to drug in Moreno et al. model.** Transmembrane potential time course during pacing with $BCL=750 ms$ for the ten Tusscher et al. model with the Moreno et al. Na^+^ current model in the presence of $5$ and $20 \mu M$ of lidocaine (A and C, respectively). The corresponding time courses for fractions of channels bound to neutral drug and in an inactivated state (orange lines), bound to neutral drug and in a non-inactivated state (yellow lines), or bound to charged drug (purple lines) are plotted in (B) and (D). Values for charged drug-bound channels were artificially increased by $0.01$ to make lines visible.

## Relative stability analysis indicates that neutral lidocaine stabilizes the inactivated state of the Na^+^ channel

Further support for our approximation that neutral lidocaine is only ever bound to inactivated channels comes from examining the relative magnitudes of the transition rate constants between inactivated and non-inactivated states when neutral lidocaine is bound. We find that in the Moreno et al. model, neutral drug-bound non-inactivated states are substantially less stable than neutral drug-bound inactivated states, meaning that following neutral drug binding to an inactivated channel, it is highly unlikely that the channel will transition to a non-inactivated state prior to drug unbinding.

To examine inactivation transition rates and stability of neutral drug bound channels (and compare them to that of non-drug bound channels), we define the “relative stability” of inactivated states to be the ratio of the steady state occupancy of non-inactivated states to that of inactivated states. For example, for the transition from the fast inactivation process to the closed states, channels recover from inactivation and inactivate with the rate constants $\alpha3$ and $\beta3$, respectively, when no drug is bound and $\alpha\_33$ and $\beta\_33$ when neutral drug is bound (see Fig 1B in the main text). Therefore, because inactivation and recovery from inactivation are in equilibrium at steady state, $\beta3*C=\alpha3*I$ and $\beta\_33*DC=\alpha\_33*DI$ where $C$ and $I$ represent the fraction of channels in the $C1$, $C2$, or $C3$ and $IF$, $IC2$, or $IC3$ states, respectively (similar for the drug bound states). Hence,

$$\frac{C}{I}=\frac{\alpha3}{\beta3} \text{and}\text{ }\frac{DC}{DI}=\frac{\alpha\_33}{\beta\_33}.$$

For the fast and slow inactivation processes from the open state, similar calculations yield

$$\frac{O}{IF}=\frac{\beta2}{\alpha2}, \frac{DO}{DIF}=\frac{\beta\_22}{\alpha\_22}, \frac{O}{IS}=\frac{\beta x}{\alpha x}, \text{and}\text{ }\frac{DO}{DIS}=\frac{\beta x2}{\alpha x2}.$$

Fig 2A displays that the ratio of closed to fast inactivated states is $265$ times smaller when neutral drug is bound ($DC/DI$, orange line) than in the drug-free case ($C/I$, blue line). Similarly, Fig 2B and C display that the ratios of open to fast inactivated states and open to slow inactivated states are $59$ times smaller when drug is bound ($DO/DIF$ and $DO/DIS$, orange lines) than in the drug-free state ($O/IF$ and $O/IS$, blue lines). In summary, following neutral lidocaine binding to an inactivated Na^+^ channel, it is highly unlikely that the channel will transition to a non-inactivated state prior to drug unbinding.


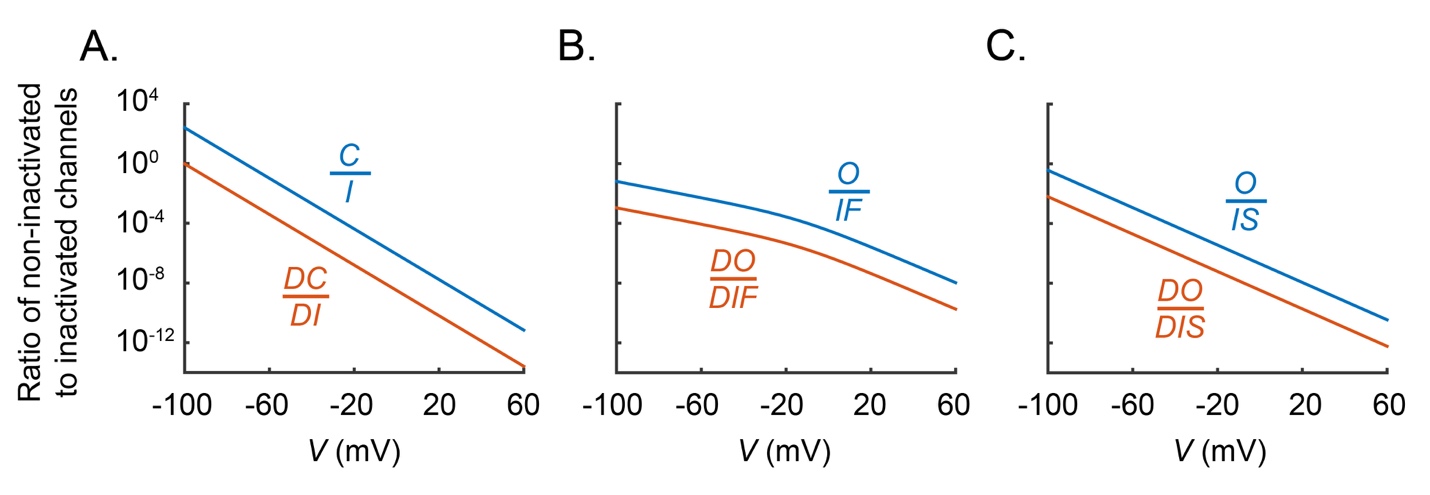


**Fig 2: Inactivation stability.** Steady state ratio of fractions of channels in non-inactivated states to inactivated states in the Moreno et al. 2011 [1] lidocaine model as a function of transmembrane potential, $V$. Lines represent the ratios of closed to fast inactivated (A), open to fast inactivated (B), and open to slow inactivated (C) states, for channels with neutral drug bound (orange lines) and without drug bound (blue lines).

# References

1. Moreno JD, Zhu ZI, Yang PC, Bankston JR, Jeng MT, Kang C, et al. A computational model to predict the effects of class I anti-arrhythmic drugs on ventricular rhythms. Sci Transl Med. 2011;3(98):98ra83. doi: 10.1126/scitranslmed.3002588. PubMed PMID: 21885405; PubMed Central PMCID: PMCPMC3328405.

2. ten Tusscher KH, Noble D, Noble PJ, Panfilov AV. A model for human ventricular tissue. American journal of physiology Heart and circulatory physiology. 2004;286(4):H1573-89. Epub 2003/12/06. doi: 10.1152/ajpheart.00794.2003. PubMed PMID: 14656705.

3. ten Tusscher KH, Panfilov AV. Alternans and spiral breakup in a human ventricular tissue model. American journal of physiology Heart and circulatory physiology. 2006;291(3):H1088-100. Epub 2006/03/28. doi: 10.1152/ajpheart.00109.2006. PubMed PMID: 16565318.
