# Supplementary material for: Rate-dependent effects of lidocaine on cardiac dynamics: Development and analysis of a low-dimensional drug-channel interaction model
Source: PLoS Comput Biol. 2021 Jun 29;17(6):e1009145. doi: 10.1371/journal.pcbi.1009145 (PMC8274935; doi:10.1371/journal.pcbi.1009145)
Supplement: S2 Appendix — (DOCX) [file pcbi.1009145.s002.docx]

1. **Exp**erimental voltage-clamp protocols

The following two subsections outline the experimental Voltage-clamp protocols used to generate the data presented in Figs 2 (drug-free data) and 3 (lidocaine data) in the main text.

## Drug-free experimental protocols for data in Fig 2 of main text

Here, we briefly describe the experimental protocols used to generate the data presented in Fig 2 of the main text, to which our low-dimensional model was fit.

Steady state availability (Fig 2A): The steady state availability data is from Fig 5B of Liu et al. [1] and was collected from HEK293 cells expressing Na^+^ channels at $22℃$. Conditioning pulses at various potentials ($V_{cond}$) were followed by a test pulse to $-10 mV$, and peak conductance was recorded. Peak conductances corresponding to each $V_{cond}$ were normalized to the peak conductance corresponding to the most negative conditioning pulse ($-130 mV$).

Steady state activation (Fig 2B): The steady state activation data is from Fig 4A of Rivolta et al. [2] and was collected from HEK293 cells expressing Na^+^ channels at $22℃$. Conditioning pulses at $-100 mV$ were followed by test pulses to varying potentials, $V_{test}$, and peak conductance was recorded. Peak conductances corresponding to each $V_{test}$ were normalized by the maximal peak conductance observed.

Time constant of inactivation (Fig 2C): (i) For hyperpolarized potentials, time to half-recovery from inactivation is from Supplementary Information of Moreno et al. 2016 [3] that was collected from HEK293 cells expressing Na^+^ channels at $22℃$. Inactivation was induced with a $-10 mV$ conditioning pulse ($100 ms$) before holding at the recovery voltage ($V_{recov}=-120 mV$, $-100 mV$, or $-80 mV$). Test pulses to $-10 mV$ for $25ms$ were applied after various recovery intervals $\left( \Delta t \right)$ and peak current recorded. We used this data to approximate the recovery interval ($t_{1/2}$) that would result in a peak conductance that is half the maximal peak conductance (i.e., following an infinite recovery interval). (ii) For depolarized potentials, time to half inactivation is from Fig 3B of Rivolta et al. [2], and was collected from HEK293 cells expressing Na^+^ channels at $22℃$. Conditioning pulses at $-100 mV$ were followed by test pulses of $40 ms$ to various potentials ($V_{test}=-35 mV$ to $20 mV$ in $5 mV$ increments). Time required for the conductance to decay to half its peak value was recorded.

Time constant of activation, $\tau_{m}$ (Fig 2D): The time constant of activation data is computed from the experimental data in Table 1 of Mitsuiye and Noma [4], which was collected from guinea-pig heart cells at $19℃$ [4]. N-bromoacetamide was used to remove Na^+^ channel inactivation. Conditioning pulses at $-100 mV$ were followed by test pulses to various potentials and current traces were recorded. Current traces of Na^+^ current activation were fit with a single exponential. We use a least squares fit to find the $\tau_{m}$ that gives the best agreement between our $m^{3}$ activation model and the Mitsuiye and Noma single exponential. The data points in Fig 2D indicate $\tau_{m}$s from the least squares fit.

It should be noted that Mitsuiye and Noma performed their experiments at $19℃$, whereas all other data in Fig 2 of the main text were collected at $22℃$. Therefore, activation time constant data from [4] was adjusted to $22℃$ using a $Q_{10}$ factor of $3$.

## Differences in data for fitting of drug-free Moreno et al. model and low-dimensional model

There are some slight differences in the data used to fit the Moreno et al. model and our Hodgkin-Huxley model. First, we fit to the activation time constant data in Fig 2D, whereas Moreno et al. used mean channel open time at $-30 mV$ [5] to constrain activation rates. Second, instead of time to half inactivation for $V=-120, -100$, and $-80 mV$, which we use for the low-dimensional model, the Moreno et al. model was fit to a time course for recovery from inactivation at $-100 mV$ to constrain the time constant of inactivation at hyperpolarized potentials. Third, the Moreno et al. model was only fit to time to half inactivation data for $V$ between $-20$ and $20 mV$, while we use all data for $V$ between $-35$ and $20 mV$ shown in Fig 2C. Finally, the Moreno et al. model was also fit to recovery from use dependent block data, which provides information about the slow inactivation processes. However, we purposefully chose the simplest Hodgkin-Huxley type Na^+^ current model, which does not include a slow inactivation gate (often referred to as a “j gate”). Therefore, we excluded the recovery from use dependent block data from our optimization.

## Lidocaine experimental protocols for data in Fig 3 of main text

All experimental data characterizing the effects of lidocaine were collected using voltage-clamp experiments performed at $22℃$ on HEK293 cells expressing only Na^+^ channels [1,5,6].

Steady state availability (Fig 3A): The steady state availability data is from Fig 5B of Liu et al. [1]. After the addition of $100 \mu M$ of lidocaine, cells were held at $-100 mV$ for $10 s$ prior to $5 s$ conditioning pulses at various potentials. Peak conductances were measured during a $25 ms$ test pulse at $-10 mV$. Peak conductance data was normalized by the peak conductance resulting from the most negative conditioning pulse ($-130 mV$).

Frequency dependence of block (Fig 3B): The frequency dependent block data is from Fig 3C of Abriel et al. [6]. After the addition of $300 \mu M$ of lidocaine, cells were paced $100$ times from $-100 mV$ to $-10 mV$ for $25 ms$ at various frequencies. The differences between the peak conductance during the first and last stimulus were calculated and then normalized by the peak conductance during the first stimulus to obtain fractional block.

Tonic block (Fig 3C): The tonic block data is from Fig 2C of Abriel et al. [6]. Cells were held at $-100 mV$ with various concentrations of lidocaine. Peak conductance was measured during a $25 ms$ test pulse to $-10 mV$. Peak conductances were then normalized to the peak conductance in the absence of drug.

Dose dependence of use-dependent block (Fig 3D): The dose dependence of use-dependent block data is from Fig 3A of Abriel et al. [6]. Cells were paced $600$ times from $-100 mV$ to $-10 mV$ for $25 ms$, at a frequency of $5 Hz$. Peak conductance during the last stimulus was normalized by the peak conductance elicited from a cell held at $-100 mV$ and then stimulated to $-10 mV$ in the absence of drug. Note: in the Fig in Abriel et al., peak conductance during the last stimulus is normalized by tonic block.

Recovery from use-dependent block (Fig 3E): The recovery from use-dependent block data is from Figs 6A in Liu et al. [5] and 1C in Moreno et al. [7] for drug-free and in the presence of $300 \mu M$ lidocaine, respectively. Cells were paced $100$ times from $-100 mV$ to $-10 mV$ for $25 ms$, at a frequency of $25 Hz$. Cells were then held at $-100 mV$ for variable intervals before a test pulse to $-10 mV$ was applied and peak conductance measured. Peak conductance was normalized by peak conductance during slow pacing at $0.033 Hz$.

# References

1. Liu H, Atkins J, Kass RS. Common molecular determinants of flecainide and lidocaine block of heart Na+ channels: evidence from experiments with neutral and quaternary flecainide analogues. J Gen Physiol. 2003;121(3):199-214. PubMed PMID: 12601084; PubMed Central PMCID: PMCPMC2217334.

2. Rivolta I, Abriel H, Tateyama M, Liu H, Memmi M, Vardas P, et al. Inherited Brugada and long QT-3 syndrome mutations of a single residue of the cardiac sodium channel confer distinct channel and clinical phenotypes. J Biol Chem. 2001;276(33):30623-30. doi: 10.1074/jbc.M104471200. PubMed PMID: 11410597.

3. Moreno JD, Lewis TJ, Clancy CE. Parameterization for In-Silico Modeling of Ion Channel Interactions with Drugs. PLoS One. 2016;11(3):e0150761. doi: 10.1371/journal.pone.0150761. PubMed PMID: 26963710; PubMed Central PMCID: PMCPMC4786197.

4. Mitsuiye T, Noma A. Quantification of exponential Na+ current activation in N-bromoacetamide-treated cardiac myocytes of guinea-pig. J Physiol. 1993;465:245-63. PubMed PMID: 8229835; PubMed Central PMCID: PMCPMC1175428.

5. Liu H, Tateyama M, Clancy CE, Abriel H, Kass RS. Channel openings are necessary but not sufficient for use-dependent block of cardiac Na(+) channels by flecainide: evidence from the analysis of disease-linked mutations. J Gen Physiol. 2002;120(1):39-51. PubMed PMID: 12084774; PubMed Central PMCID: PMCPMC2311398.

6. Abriel H, Wehrens XH, Benhorin J, Kerem B, Kass RS. Molecular pharmacology of the sodium channel mutation D1790G linked to the long-QT syndrome. Circulation. 2000;102(8):921-5. PubMed PMID: 10952963.

7. Moreno JD, Zhu ZI, Yang PC, Bankston JR, Jeng MT, Kang C, et al. A computational model to predict the effects of class I anti-arrhythmic drugs on ventricular rhythms. Sci Transl Med. 2011;3(98):98ra83. doi: 10.1126/scitranslmed.3002588. PubMed PMID: 21885405; PubMed Central PMCID: PMCPMC3328405.
