# Supplementary material for: Rate-dependent effects of lidocaine on cardiac dynamics: Development and analysis of a low-dimensional drug-channel interaction model
Source: PLoS Comput Biol. 2021 Jun 29;17(6):e1009145. doi: 10.1371/journal.pcbi.1009145 (PMC8274935; doi:10.1371/journal.pcbi.1009145)
Supplement: S3 Appendix — (DOCX) [file pcbi.1009145.s003.docx]

# Low-dimensional model under predicts tonic block at $\boldsymbol{-100 mV}$ (but not at the more physiological resting potential of $\boldsymbol{-85 mV}$)

Our low-dimensional model under predicts the level of tonic block at $-100 mV$ (Fig 3C of the main text). On the other hand, the Moreno et al. model accurately reproduces the experimental tonic block data. Here, we use the Moreno et al. model to explain why our model under predicts tonic block at $-100 mV$. We also demonstrate that our model agrees with the Moreno et al. model at a more physiological resting potential ($-85 mV$), and therefore presumably our model replicates the physiological level of tonic block.

The disparity between the experimental data and our low-dimensional model’s predictions of tonic block (Fig 3C of the main text) can be understood by considering the $K_{d}$’s (ratios of drug unbinding rate to drug binding rate) for lidocaine-Na^+^ channel interactions in various conformational states. As stated in Section 2.2, neutral lidocaine binds to inactivated Na^+^ channels with a $K_{d}$ of $6.8 \mu M$ [1, 2]. However, at $V=-100 mV$, very few Na^+^ channels are inactivated. Therefore, because our low-dimensional model includes only the neutral form of lidocaine binding to inactivated channels, the overall binding and unbinding rates at $V=-100 mV$ are $\left( 1-h_{\infty}\left( -100 \right) \right)k_{on}$ and $k_{off}$, respectively, and the $K_{d}$ is

$$K_{d}\left( -100 \right)=\frac{k_{off}}{\left( 1-h_{\infty}\left( -100 \right) \right)k_{on}}=1100 \mu M.$$

This $K_{d}$ at $-100 mV$ is the same order of magnitude as the $K_{d}$’s for neutral lidocaine binding to non-inactivated channels ($1800$ and $400 \mu M$ for closed and open channels, respectively) and charged lidocaine binding to non-inactivated channels at $-100 mV$ ($5000 \mu M$) at $22 ℃$ [1-4]. Hence, at $-100 mV$ lidocaine binding is not dominated by the neutral form binding to inactivated channels, as we assume in our low-dimensional model.

The Moreno et al. model replicates tonic block data better than our model (Fig 1A) because, unlike our model, it includes the effects of charged lidocaine and the ability of lidocaine to bind to non-inactivated channels. Specifically, predicted tonic block decreases (i.e., normalized peak conductance increases) in the Moreno et al. model when the effects of charged lidocaine are removed from the model (dashed yellow line), and decreases further when neutral drug-bound, non-inactivated states are also removed from the model (dashed purple line).


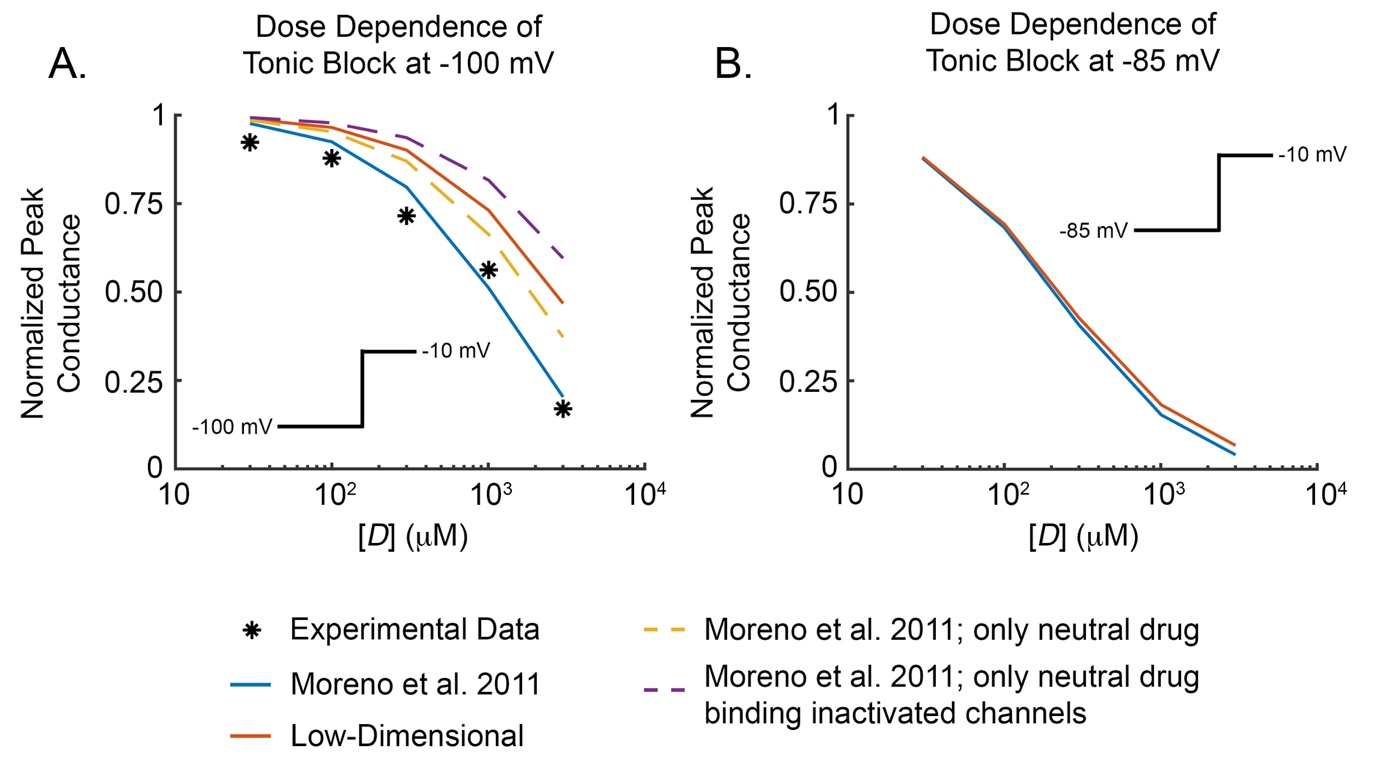


**Fig 1: Tonic block with holding potentials of** $\boldsymbol{-100 mV}$ **(A) and** $\boldsymbol{-85 mV}$ **(B).** Experimental data (asterisks), Moreno et al. 2011 model (blue lines), low-dimensional model (orange lines), Moreno et al. 2011 model with only neutral drug effects (dashed yellow line), and Moreno et al. 2011 model with only neutral drug binding to inactivated channels (dashed purple line).

However, predictions for tonic block at a more physiological resting potential ($-85 mV$) from our low-dimensional model and the Moreno et al. model nearly overlap (Fig 1B). In fact, the largest observed difference in normalized peak conductance is $0.031$ at a lidocaine concentration of $1000 \mu M$. The improved agreement in tonic block at less hyperpolarized potentials is due to the greater fraction of inactivated Na^+^ channels, which causes lidocaine binding to be dominated by the neutral form binding to inactivated channels.

# References

1. Moreno JD, Zhu ZI, Yang PC, Bankston JR, Jeng MT, Kang C, et al. A computational model to predict the effects of class I anti-arrhythmic drugs on ventricular rhythms. Sci Transl Med. 2011;3(98):98ra83. doi: 10.1126/scitranslmed.3002588. PubMed PMID: 21885405; PubMed Central PMCID: PMCPMC3328405.

2. Liu H, Atkins J, Kass RS. Common molecular determinants of flecainide and lidocaine block of heart Na+ channels: evidence from experiments with neutral and quaternary flecainide analogues. J Gen Physiol. 2003;121(3):199-214. PubMed PMID: 12601084; PubMed Central PMCID: PMCPMC2217334.

3. Bennett PB, Valenzuela C, Chen LQ, Kallen RG. On the molecular nature of the lidocaine receptor of cardiac Na+ channels. Modification of block by alterations in the alpha-subunit III-IV interdomain. Circ Res. 1995;77(3):584-92. Epub 1995/09/01. PubMed PMID: 7641328.

4. Abriel H, Wehrens XH, Benhorin J, Kerem B, Kass RS. Molecular pharmacology of the sodium channel mutation D1790G linked to the long-QT syndrome. Circulation. 2000;102(8):921-5. PubMed PMID: 10952963.
