# Supplementary material for: Rate-dependent effects of lidocaine on cardiac dynamics: Development and analysis of a low-dimensional drug-channel interaction model
Source: PLoS Comput Biol. 2021 Jun 29;17(6):e1009145. doi: 10.1371/journal.pcbi.1009145 (PMC8274935; doi:10.1371/journal.pcbi.1009145)
Supplement: S4 Appendix — (DOCX) [file pcbi.1009145.s004.docx]

# Peak upstroke and conduction velocity in modified ten Tusscher et al. models

Fig 4 of the main text shows the dependencies of normalized peak upstroke velocity and conduction velocity on BCL in the ten Tusscher et al. human ventricular myocyte model [1,2] with the Moreno et al. [3] and our low-dimensional models of the Na^+^ current. To construct the modified ten Tusscher et al. models, we replace the ten Tusscher fast Na^+^ conductance with either the Moreno et al. model or our low-dimensional model. As the ten Tusscher et al. model is parameterized for $37℃$, we use the $37℃$ parameterizations of the Moreno et al. and low-dimensional models. The maximal Na^+^ conductance per capacitance in the Moreno et al. model is $15 nS/pF$ [3], and the maximal conductance per capacitance of the low-dimensional model is set to $20 nS/pF$, so that conduction velocity at a BCL of $1000 ms$ with no drug is the same in both models.

For each BCL, peak upstroke velocity is recorded after the model cell is paced $500$ times with stimuli of amplitude $-80 pA/pF$ and duration $1 ms$.

To measure conduction velocity, waves are generated by depolarizing the distal $0.1 cm$ of a $1.1 cm$ long cable to $0 mV$. $500$ waves are generated at a BCL of $1000 ms$ and the BCL is subsequently decreased to $300 ms$ in increments of $50 ms$ with $10$ stimuli applied at each BCL. Conduction velocity is recorded for the last two stimuli of each pacing rate at the point $0.6cm$ from the distal end.

Normalized upstroke and conduction velocities for the modified ten Tusscher et al. models with $5 \mu M$ and $20 \mu M$ of lidocaine are presented in Fig 4 of the main text; here, the corresponding non-normalized upstroke and conduction velocities are displayed in Fig 1 along with the results for the drug-free cases. The maximal Na^+^ conductance in the low-dimensional model (conductance per capacitance of $20 nS/pF$) is relatively high compared to the Moreno et al. model at (conductance per capacitance of $15 nS/pF$), causing peak upstroke velocity to be greater in the low-dimensional model than in the Moreno et al. model. Note that the rate-dependencies of peak upstroke velocity and conduction velocity are slightly different in the two drug-free models, with upstroke and conduction velocities decreasing more at short BCL in the Moreno et al. model. The disparities in the rate-dependencies of the drug-free models are due to the differences in formulation of the drug-free Na^+^ current models, which are also responsible for some of the discrepancy in rate-dependence of the models in the presence of lidocaine. Therefore, in the main text, we present upstroke and conduction velocities normalized by peak upstroke and conduction velocities of the corresponding drug-free model, so as to focus on the rate-dependent effects of lidocaine and not the combined rate-dependence of lidocaine and the underlying Na^+^ current.


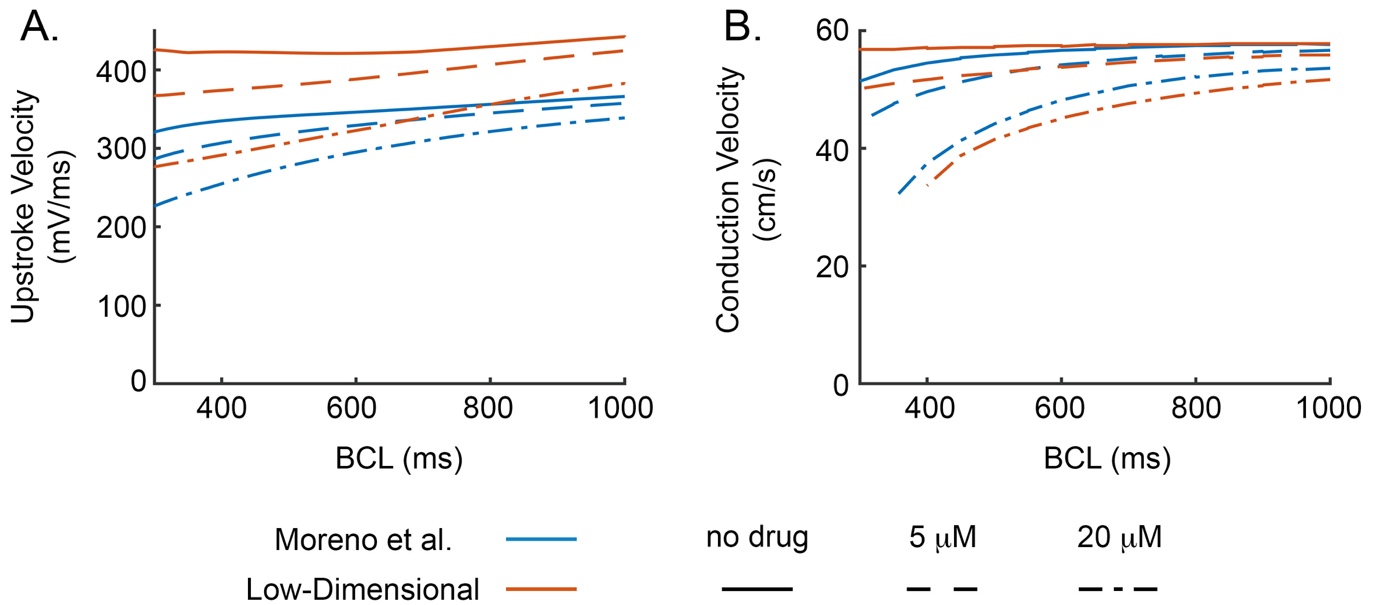


**Fig 1: Rate-Dependent Effects of Lidocaine.** Peak upstroke velocity (A) and conduction velocity (B) plotted against BCL for the ten Tusscher et al. human ventricular myocyte model [1, 2] with the Moreno et al. model (blue lines) or low-dimensional model (orange lines) of the Na^+^ current{ten Tusscher, 2004 #5}. Peak upstroke and conduction velocities in the absence of drug (solid lines), and in the presence of $5 \mu M$ (dashed lines) and $20 \mu M$ (dot-dashed lines) concentrations of lidocaine are plotted.

In addition to conduction velocity under steady pacing, we examine the effect of lidocaine on conduction velocity following a premature beat. To measure conduction velocity from a premature beat, a single premature beat was applied after steady pacing $500$ times at a BCL of $1000 ms$. The interval between the stimulus of the final regular beat and the premature beat is listed as BCL in Figs 2 and 3. We see that for both the Moreno et al. model and our low-dimensional model, conduction velocity following a premature beat is very similar to conduction velocity at steady pacing for all drug concentrations. The only exception being that we see stronger restitution properties at short BCL following a premature beat than in steady pacing for both models.

#
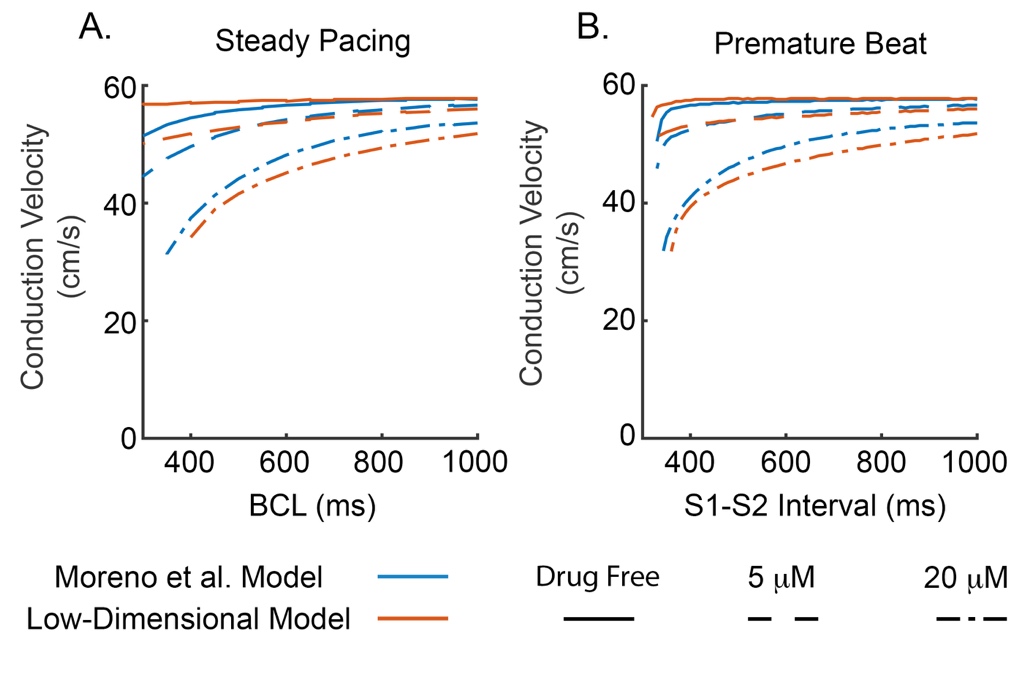


**Fig 2:** Comparison of CV vs. pacing period in 2 models for the case of steady pacing (left panel) and following a single premature beat (right panel). For steady pacing, $500$ waves are generated at a BCL of $1000 ms$ and the BCL is subsequently decreased to $300 ms$ in increments of $50 ms$ with $10$ stimuli applied at each BCL. For CV resultant from a premature beat, a single premature beat is applied after pacing $500$ times at a BCL of $1000 ms$. CV is plotted against the interval between the last steady pacing beat (S1) and the premature beat (S2).


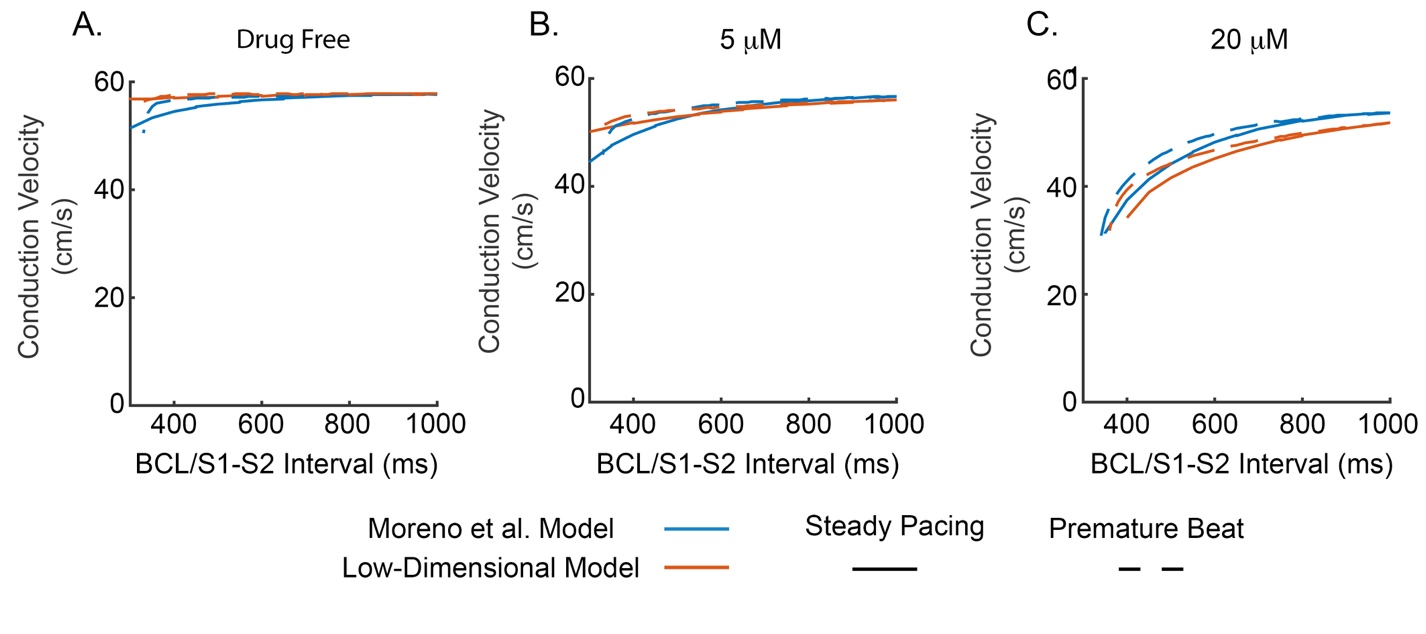


**Fig 3:** Comparison of CV vs. pacing period during steady pacing (solid lines) and following a single premature beat (dashed lines) for the 3 drug concentrations. For steady pacing, $500$ waves are generated at a BCL of $1000 ms$ and the BCL is subsequently decreased to $300 ms$ in increments of $50 ms$ with $10$ stimuli applied at each BCL. For CV resultant from a premature beat, a single premature beat is applied after pacing $500$ times at a BCL of $1000 ms$. CV is plotted against the interval between the last steady pacing beat (S1) and the premature beat (S2).

# References

1. ten Tusscher KH, Noble D, Noble PJ, Panfilov AV. A model for human ventricular tissue. American journal of physiology Heart and circulatory physiology. 2004;286(4):H1573-89. Epub 2003/12/06. doi: 10.1152/ajpheart.00794.2003. PubMed PMID: 14656705.

2. ten Tusscher KH, Panfilov AV. Alternans and spiral breakup in a human ventricular tissue model. American journal of physiology Heart and circulatory physiology. 2006;291(3):H1088-100. Epub 2006/03/28. doi: 10.1152/ajpheart.00109.2006. PubMed PMID: 16565318.

3. Moreno JD, Zhu ZI, Yang PC, Bankston JR, Jeng MT, Kang C, et al. A computational model to predict the effects of class I anti-arrhythmic drugs on ventricular rhythms. Sci Transl Med. 2011;3(98):98ra83. doi: 10.1126/scitranslmed.3002588. PubMed PMID: 21885405; PubMed Central PMCID: PMCPMC3328405.
