# Supplementary material for: Rate-dependent effects of lidocaine on cardiac dynamics: Development and analysis of a low-dimensional drug-channel interaction model
Source: PLoS Comput Biol. 2021 Jun 29;17(6):e1009145. doi: 10.1371/journal.pcbi.1009145 (PMC8274935; doi:10.1371/journal.pcbi.1009145)
Supplement: S5 Appendix — (DOCX) [file pcbi.1009145.s005.docx]

# Dependence of lidocaine binding on physiological properties

In Section 3.2.2 of the main text, we derive an expression for the fraction of channels bound to neutral lidocaine during the upstroke, $b^{*}$, that captures the parametric dependence on BCL, action potential duration restitution properties, plateau potential, diastolic potential, channel inactivation kinetics, and drug concentration and binding rates. We then use this expression for $b^{*}$ and the partial derivatives of $b^{*}$ to examine how lidocaine binding depends on the physiological properties of restitution curve and transmembrane potential during the AP and DI. Here, we derive the partial derivatives of $b^{*}$.

The expression for $b^{*}$ is

$$b^{*}=\left[ \frac{1-D}{1-AD} \right]b_{\infty}\left( V_{DI} \right)+\left[ \frac{\left( 1-A \right)D}{1-AD} \right]b_{\infty}\left( V_{AP} \right),$$

where

$$A=e^{- \frac{APD}{\tau_{b}\left( V_{AP} \right)}}, D=e^{- \frac{DI}{\tau_{b}\left( V_{DI} \right)}}$$

and

$$\tau_{b}\left( V \right)=\frac{1}{\left( 1-h_{\infty}\left( V \right) \right)\left[ D \right]k_{on}+k_{off}}, b_{\infty}\left( V \right)=\frac{\left( 1-h_{\infty}\left( V \right) \right)\left[ D \right]k_{on}}{\left( 1-h_{\infty}\left( V \right) \right)\left[ D \right]k_{on}+k_{off}}.$$

Also, we define $APD=f\left( BCL \right)$ to be the restitution curve and note that $DI=BCL-APD=BCL-f\left( BCL \right)$.

## Partial derivative of $\boldsymbol{b}^{\boldsymbol{*}}$ with respect to BCL

$$\frac{\partial b^{*}}{\partial BCL}=\left[ -\frac{\partial D}{\partial BCL}\left( 1-AD \right)^{-1}+\left( 1-D \right)\left( 1-AD \right)^{-2}\left( A\frac{\partial D}{\partial BCL}+D\frac{\partial A}{\partial BCL} \right) \right]b_{\infty}\left( V_{DI} \right)+\left[ \frac{\partial D}{\partial BCL}\left( 1-A \right)\left( 1-AD \right)^{-1}-\frac{\partial A}{\partial BCL}D\left( 1-AD \right)^{-1}+\left( 1-A \right)D\left( 1-AD \right)^{-2}\left( A\frac{\partial D}{\partial BCL}+D\frac{\partial A}{\partial BCL} \right) \right]b_{\infty}\left( V_{AP} \right)$$

$$=\left[ -\frac{\partial D}{\partial BCL}\left( 1-AD \right)+\left( 1-D \right)\left( A\frac{\partial D}{\partial BCL}+D\frac{\partial A}{\partial BCL} \right) \right]\frac{b_{\infty}\left( V_{DI} \right)}{\left( 1-AD \right)^{2}}+\left[ \frac{\partial D}{\partial BCL}\left( 1-A \right)\left( 1-AD \right)-\frac{\partial A}{\partial BCL}D\left( 1-AD \right)+\left( 1-A \right)D\left( A\frac{\partial D}{\partial BCL}+D\frac{\partial A}{\partial BCL} \right) \right]\frac{b_{\infty}\left( V_{AP} \right)}{\left( 1-AD \right)^{2}}$$

$$=\left[ \left( A-1 \right)\frac{\partial D}{\partial BCL}+D\left( 1-D \right)\frac{\partial A}{\partial BCL} \right]\frac{b_{\infty}\left( V_{DI} \right)}{\left( 1-AD \right)^{2}}+\left[ \left( 1-A \right)\frac{\partial D}{\partial BCL}+D\left( D-1 \right)\frac{\partial A}{\partial BCL} \right]\frac{b_{\infty}\left( V_{AP} \right)}{\left( 1-AD \right)^{2}}$$

$$=\frac{1}{\left( 1-AD \right)^{2}}\left[ b_{\infty}\left( V_{AP} \right)-b_{\infty}\left( V_{DI} \right) \right]\left[ \left( 1-A \right)\frac{\partial D}{\partial BCL}-D\left( 1-D \right)\frac{\partial A}{\partial BCL} \right].$$

We note that

$$\frac{\partial D}{\partial BCL}=\frac{\partial}{\partial BCL}\left( e^{- \frac{DI}{\tau_{b}\left( V_{DI} \right)}} \right)$$

$$=-\frac{1}{\tau_{b}\left( V_{DI} \right)}\frac{dDI}{dBCL}e^{- \frac{DI}{\tau_{b}\left( V_{DI} \right)}}$$

$$=-\frac{1}{\tau_{b}\left( V_{DI} \right)}\left( 1-\frac{df\left( BCL \right)}{dBCL} \right)e^{- \frac{DI}{\tau_{b}\left( V_{DI} \right)}}$$

$$=-\frac{1}{\tau_{b}\left( V_{DI} \right)}\left( 1-\frac{df\left( BCL \right)}{dBCL} \right)D$$

and

$$\frac{\partial A}{\partial BCL}=\frac{\partial}{\partial BCL}\left( e^{- \frac{APD}{\tau_{b}\left( V_{AP} \right)}} \right)$$

$$=-\frac{1}{\tau_{b}\left( V_{AP} \right)}\frac{dAPD}{dBCL}e^{- \frac{APD}{\tau_{b}\left( V_{AP} \right)}}$$

$$=-\frac{1}{\tau_{b}\left( V_{AP} \right)}\frac{df\left( BCL \right)}{dBCL}e^{- \frac{APD}{\tau_{b}\left( V_{AP} \right)}}$$

$$=-\frac{1}{\tau_{b}\left( V_{AP} \right)}\frac{df\left( BCL \right)}{dBCL}A.$$

Inserting the expressions for $\frac{\partial D}{\partial BCL}$ and $\frac{\partial A}{\partial BCL}$ into the expression for $\frac{\partial b^{*}}{\partial BCL}$ produces

$$\frac{\partial b^{*}}{\partial BCL}=\frac{b_{\infty}\left( V_{AP} \right)-b_{\infty}\left( V_{DI} \right)}{\left( 1-AD \right)^{2}}\left[ \frac{\left( 1-D \right)AD}{\tau_{b}\left( V_{AP} \right)}\frac{df\left( BCL \right)}{dBCL}-\frac{\left( 1-A \right)D}{\tau_{b}\left( V_{DI} \right)}\left( 1-\frac{df\left( BCL \right)}{dBCL} \right) \right],$$

which is Equation $(6)$ in the main text.

## Partial derivative of $\boldsymbol{b}^{\boldsymbol{*}}$ with respect to $\boldsymbol{V}_{\boldsymbol{AP}}$

$$\frac{\partial b^{*}}{\partial V_{AP}}=\left( 1-D \right)\left( 1-AD \right)^{-2}\frac{\partial A}{\partial V_{AP}}Db_{\infty}\left( V_{DI} \right)+\left[ -\frac{\partial A}{\partial V_{AP}}D\left( 1-AD \right)^{-1}+\left( 1-A \right)D\left( 1-AD \right)^{-2}\frac{\partial A}{\partial V_{AP}}D \right]b_{\infty}\left( V_{AP} \right)+\left( 1-A \right)D\left( 1-AD \right)^{-1}\frac{\partial b_{\infty}\left( V_{AP} \right)}{\partial V_{AP}}$$

$$=\frac{1}{\left( 1-AD \right)^{2}}\left[ \left( 1-D \right)Db_{\infty}\left( V_{DI} \right)+\left[ -\left( D-AD^{2} \right)+\left( D^{2}-AD^{2} \right) \right]b_{\infty}\left( V_{AP} \right) \right]\frac{\partial A}{\partial V_{AP}}+\left( 1-A \right)D\left( 1-AD \right)^{-1}\frac{\partial b_{\infty}\left( V_{AP} \right)}{\partial V_{AP}}$$

$$=\frac{1}{\left( 1-AD \right)^{2}}\left[ \left( 1-D \right)Db_{\infty}\left( V_{DI} \right)+\left( D-1 \right)Db_{\infty}\left( V_{AP} \right) \right]\frac{\partial A}{\partial V_{AP}}+\left( 1-A \right)D\left( 1-AD \right)^{-1}\frac{\partial b_{\infty}\left( V_{AP} \right)}{\partial V_{AP}}$$

$$=\frac{\left( 1-D \right)D}{\left( 1-AD \right)^{2}}\left[ b_{\infty}\left( V_{DI} \right)-b_{\infty}\left( V_{AP} \right) \right]\frac{\partial A}{\partial V_{AP}}+\frac{\left( 1-A \right)D}{1-AD}\frac{\partial b_{\infty}\left( V_{AP} \right)}{\partial V_{AP}}.$$

We note that

$$A=e^{- \frac{APD}{\tau_{b}\left( V_{AP} \right)}}=e^{- APD\left[ \left( 1-h_{\infty}\left( V_{AP} \right) \right)\left[ D \right]k_{on}+k_{off} \right]}$$

and

$$b_{\infty}\left( V_{AP} \right)=\frac{\left( 1-h_{\infty}\left( V_{AP} \right) \right)\left[ D \right]k_{on}}{\left( 1-h_{\infty}\left( V_{AP} \right) \right)\left[ D \right]k_{on}+k_{off}},$$

therefore,

$$\frac{\partial A}{\partial V_{AP}}=\frac{\partial}{\partial V_{AP}}\left( e^{- APD\left[ \left( 1-h_{\infty}\left( V_{AP} \right) \right)\left[ D \right]k_{on}+k_{off} \right]} \right)$$

$$=APD\frac{dh_{\infty}\left( V_{AP} \right)}{dV_{AP}}\left[ D \right]k_{on}e^{- APD\left[ \left( 1-h_{\infty}\left( V_{AP} \right) \right)\left[ D \right]k_{on}+k_{off} \right]}$$

$$=APD\left[ D \right]k_{on}\frac{dh_{\infty}\left( V_{AP} \right)}{dV_{AP}}A$$

and

$$\frac{\partial b_{\infty}\left( V_{AP} \right)}{\partial V_{AP}}=-\frac{dh_{\infty}\left( V_{AP} \right)}{dV_{AP}}\frac{\left[ D \right]k_{on}}{\left( 1-h_{\infty}\left( V_{AP} \right) \right)\left[ D \right]k_{on}+k_{off}}+\left( 1-h_{\infty}\left( V_{AP} \right) \right)\left[ D \right]k_{on}\left( \left( 1-h_{\infty}\left( V_{AP} \right) \right)\left[ D \right]k_{on}+k_{off} \right)^{-2}\frac{dh_{\infty}\left( V_{AP} \right)}{dV_{AP}}\left[ D \right]k_{on}$$

$$=-\tau_{b}\left( V_{AP} \right)\left[ D \right]k_{on}\frac{dh_{\infty}\left( V_{AP} \right)}{dV_{AP}}+b_{\infty}\left( V_{AP} \right)\tau_{b}\left( V_{AP} \right)\left[ D \right]k_{on}\frac{dh_{\infty}\left( V_{AP} \right)}{dV_{AP}}$$

$$=\tau_{b}\left( V_{AP} \right)\left[ D \right]k_{on}\left( b_{\infty}\left( V_{AP} \right)-1 \right)\frac{dh_{\infty}\left( V_{AP} \right)}{dV_{AP}}.$$

Inserting the expressions for $\frac{\partial A}{\partial V_{AP}}$ and $\frac{\partial b_{\infty}\left( V_{AP} \right)}{\partial V_{AP}}$ into the expression for $\frac{\partial b^{*}}{\partial V_{AP}}$ produces

$$\frac{\partial b^{*}}{\partial V_{AP}}=\frac{\left( 1-D \right)AD}{\left( 1-AD \right)^{2}}\left[ b_{\infty}\left( V_{DI} \right)-b_{\infty}\left( V_{AP} \right) \right]APD\left[ D \right]k_{on}\frac{dh_{\infty}\left( V_{AP} \right)}{dV_{AP}}+\frac{\left( 1-A \right)D\left( 1-AD \right)}{\left( 1-AD \right)^{2}}\tau_{b}\left( V_{AP} \right)\left[ D \right]k_{on}\left[ b_{\infty}\left( V_{AP} \right)-1 \right]\frac{dh_{\infty}\left( V_{AP} \right)}{dV_{AP}}$$

$$=\frac{D\left[ D \right]k_{on}}{\left( 1-AD \right)^{2}}\left[ \left( 1-D \right)A\left[ b_{\infty}\left( V_{AP} \right)-b_{\infty}\left( V_{DI} \right) \right]APD+\left( 1-A \right)\left( 1-AD \right)\tau_{b}\left( V_{AP} \right)\left[ 1-b_{\infty}\left( V_{AP} \right) \right] \right]\frac{d\left( 1-h_{\infty}\left( V_{AP} \right) \right)}{dV_{AP}} =\xi\left( V_{AP} \right) \frac{d\left( 1-h_{\infty}\left( V_{AP} \right) \right)}{dV_{AP}},$$

where

$$\xi\left( V_{AP} \right)=\frac{D\tau_{b}\left( V_{AP} \right)\left[ D \right]k_{on}}{\left( 1-AD \right)^{2}}\left[ A\left( 1-D \right)\frac{APD}{\tau_{b}\left( V_{AP} \right)}\left[ b_{\infty}\left( V_{AP} \right)-b_{\infty}\left( V_{DI} \right) \right]+\left( 1-A \right)\left( 1-AD \right)\left[ 1-b_{\infty}\left( V_{AP} \right) \right] \right].$$

Note that $A, D<1$, and $\left( 1-A \right), \left( 1-D \right),$ $\left( 1-AD \right)>0$, and $1>b_{\infty}\left( V_{AP} \right)>b_{\infty}\left( V_{DI} \right)>0$, thus $\xi\left( V_{AP} \right)>0.$ Note further that $\left( 1-A \right), \left( 1-D \right)\leq\left( 1-AD \right)$ and $\left( 1-A \right)\geq\frac{APD}{\tau_{b}\left( V_{AP} \right)}A$ because $1-e^{-x}\geq xe^{-x}$ for all $x\geq0$, thus

$$\xi\left( V_{AP} \right)<\frac{D\tau_{b}\left( V_{AP} \right)\left[ D \right]k_{on}}{\left( 1-AD \right)^{2}}\left[ \left( 1-A \right)\left( 1-AD \right)\left[ 1-b_{\infty}\left( V_{DI} \right) \right]+\left( 1-A \right)\left( 1-AD \right)\left[ 1-b_{\infty}\left( V_{AP} \right) \right] \right]$$

$$<\frac{D}{\left( 1-AD \right)}\left[ \left( 1-AD \right)\left[ 1-b_{\infty}\left( V_{DI} \right) \right]+\left( 1-AD \right)\left[ 1-b_{\infty}\left( V_{DI} \right) \right] \right]\tau_{b}\left( V_{AP} \right)\left[ D \right]k_{on}$$

$$=2D\left[ 1-b_{\infty}\left( V_{DI} \right) \right] \frac{\left[ D \right]k_{on}}{\left( 1-h_{\infty}\left( V_{AP} \right) \right)\left[ D \right]k_{on}+k_{off}}$$

$$<2\frac{\left[ D \right]k_{on}}{k_{off}}=2\frac{\left[ D \right]}{K_{d}}.$$

For the parameters considered here, $K_{d}=6.8 \mu M$, and at clinical concentrations, the concentration of neutral lidocaine is $\left[ D \right]<8 \mu M$. Hence, $0<\xi\left( V_{AP} \right)<2.4$.

Therefore, because steady state inactivation is saturated above $-50 mV$ (i.e., $\frac{d\left( 1-h_{\infty}\left( V_{AP} \right) \right)}{dV_{AP}}\leq4.5\times{10}^{-3} mV^{-1}$ for $V_{AP}>-50 mV$ at $37℃$), $b^{*}$ is insensitive to changes in $V_{AP}$ (i.e., $\frac{\partial b^{*}}{\partial V_{AP}}\approx0$).

## Partial derivative of $\boldsymbol{b}^{\boldsymbol{*}}$ with respect to $\boldsymbol{V}_{\boldsymbol{DI}}$

$$\frac{\partial b^{*}}{\partial V_{DI}}=\left[ -\frac{\partial D}{\partial V_{DI}}\left( 1-AD \right)^{-1}+\left( 1-D \right)\left( 1-AD \right)^{-2}A\frac{\partial D}{\partial V_{DI}} \right]b_{\infty}\left( V_{DI} \right)+\frac{1-D}{1-AD}\frac{\partial b_{\infty}\left( V_{DI} \right)}{\partial V_{DI}}+\left[ \left( 1-A \right)\left( 1-AD \right)^{-1}\frac{\partial D}{\partial V_{DI}}+\left( 1-A \right)D\left( 1-AD \right)^{-2}A\frac{\partial D}{\partial V_{DI}} \right]b_{\infty}\left( V_{AP} \right)$$

$$=\frac{1}{\left( 1-AD \right)^{2}}\left[ \left[ -\left( 1-AD \right)+A\left( 1-D \right) \right]b_{\infty}\left( V_{DI} \right)+\left[ \left( 1-A \right)\left( 1-AD \right)+\left( 1-A \right)AD \right]b_{\infty}\left( V_{AP} \right) \right]\frac{\partial D}{\partial V_{DI}}+\frac{1-D}{1-AD}\frac{\partial b_{\infty}\left( V_{DI} \right)}{\partial V_{DI}}$$

$$=\frac{1}{\left( 1-AD \right)^{2}}\left[ \left( A-1 \right)b_{\infty}\left( V_{DI} \right)+\left( 1-A \right)b_{\infty}\left( V_{AP} \right) \right]\frac{\partial D}{\partial V_{DI}}+\frac{1-D}{1-AD}\frac{\partial b_{\infty}\left( V_{DI} \right)}{\partial V_{DI}}$$

$$=\frac{1-A}{\left( 1-AD \right)^{2}}\left( b_{\infty}\left( V_{AP} \right)-b_{\infty}\left( V_{DI} \right) \right)\frac{\partial D}{\partial V_{DI}}+\frac{1-D}{1-AD}\frac{\partial b_{\infty}\left( V_{DI} \right)}{\partial V_{DI}}.$$

We note that similar to $A$ and $b_{\infty}\left( V_{AP} \right)$,

$$D=e^{- \frac{DI}{\tau_{b}\left( V_{DI} \right)}}=e^{- DI\left[ \left( 1-h_{\infty}\left( V_{DI} \right) \right)\left[ D \right]k_{on}+k_{off} \right]}$$

and

$$b_{\infty}\left( V_{DI} \right)=\frac{\left( 1-h_{\infty}\left( V_{DI} \right) \right)\left[ D \right]k_{on}}{\left( 1-h_{\infty}\left( V_{DI} \right) \right)\left[ D \right]k_{on}+k_{off}} '$$

so by the calculations done in Section S5.2,

$$\frac{\partial D}{\partial V_{DI}}=DI\left[ D \right]k_{on}\frac{dh_{\infty}\left( V_{DI} \right)}{dV_{DI}}D$$

and

$$\frac{\partial b_{\infty}\left( V_{DI} \right)}{\partial V_{DI}}=\tau_{b}\left( V_{DI} \right)\left[ D \right]k_{on}\left( b_{\infty}\left( V_{DI} \right)-1 \right)\frac{dh_{\infty}\left( V_{DI} \right)}{dV_{DI}}.$$

Inserting the expressions for $\frac{\partial D}{\partial V_{DI}}$ and $\frac{\partial b_{\infty}\left( V_{DI} \right)}{\partial V_{DI}}$ into the expression for $\frac{\partial b^{*}}{\partial V_{DI}}$ produces

$$\frac{\partial b^{*}}{\partial V_{DI}}=\frac{\left( 1-A \right)D}{\left( 1-AD \right)^{2}}\left( b_{\infty}\left( V_{AP} \right)-b_{\infty}\left( V_{DI} \right) \right)DI\left[ D \right]k_{on}\frac{dh_{\infty}\left( V_{DI} \right)}{dV_{DI}}+\frac{1-D}{1-AD}\tau_{b}\left( V_{DI} \right)\left[ D \right]k_{on}\left( b_{\infty}\left( V_{DI} \right)-1 \right)\frac{dh_{\infty}\left( V_{DI} \right)}{dV_{DI}}$$

$$=\frac{\left[ D \right]k_{on}}{\left( 1-AD \right)^{2}}\left[ \left( 1-A \right)D\left( b_{\infty}\left( V_{AP} \right)-b_{\infty}\left( V_{DI} \right) \right)DI+\left( 1-AD \right)\left( 1-D \right)\tau_{b}\left( V_{DI} \right)\left( b_{\infty}\left( V_{DI} \right)-1 \right) \right]\frac{dh_{\infty}\left( V_{DI} \right)}{dV_{DI}}$$

$$=\frac{\tau_{b}\left( V_{DI} \right)\left[ D \right]k_{on}}{\left( 1-AD \right)^{2}}\left[ \left( 1-A \right)D\frac{-DI}{\tau_{b}\left( V_{DI} \right)}\left( b_{\infty}\left( V_{AP} \right)-b_{\infty}\left( V_{DI} \right) \right)+\left( 1-AD \right)\left( 1-D \right)\left( 1-b_{\infty}\left( V_{DI} \right) \right) \right]\frac{d\left( 1-h_{\infty}\left( V_{DI} \right) \right)}{dV_{DI}}$$

$$=\gamma\left( V_{DI} \right)\frac{d\left( 1-h_{\infty}\left( V_{DI} \right) \right)}{dV_{DI}},$$

where

$$\gamma\left( V_{DI} \right)=\frac{\tau_{b}\left( V_{DI} \right)\left[ D \right]k_{on}}{\left( 1-AD \right)^{2}}\left[ \left( 1-A \right)D\frac{-DI}{\tau_{b}\left( V_{DI} \right)}\left( b_{\infty}\left( V_{AP} \right)-b_{\infty}\left( V_{DI} \right) \right)+\left( 1-AD \right)\left( 1-D \right)\left( 1-b_{\infty}\left( V_{DI} \right) \right) \right]$$

Note that $0<1-A<1-AD$, and $0<b_{\infty}\left( V_{AP} \right)-b_{\infty}\left( V_{DI} \right)<1-b_{\infty}\left( V_{DI} \right)$, and $0<\frac{DI}{\tau_{b}\left( V_{DI} \right)}D\leq1-D$ because $xe^{-x}\leq1-e^{-x}$ for all $x\geq0$. Thus, $\left( 1-A \right)D\frac{DI}{\tau_{b}\left( V_{DI} \right)}\left( b_{\infty}\left( V_{AP} \right)-b_{\infty}\left( V_{DI} \right) \right)<\left( 1-AD \right)\left( 1-D \right)\left( 1-b_{\infty}\left( V_{DI} \right) \right)$, and as a result $\gamma\left( V_{DI} \right)>0$. Additionally, note that $0<1-D<1-AD$, thus

$$\gamma\left( V_{DI} \right)<\frac{\tau_{b}\left( V_{DI} \right)\left[ D \right]k_{on}}{\left( 1-AD \right)^{2}}\left[ \left( 1-AD \right)\left( 1-D \right)\left( 1-b_{\infty}\left( V_{DI} \right) \right) \right]$$

$$<\frac{\tau_{b}\left( V_{DI} \right)\left[ D \right]k_{on}}{\left( 1-AD \right)^{2}}\left( 1-AD \right)^{2}$$

$$=\frac{\left[ D \right]k_{on}}{\left( 1-h_{\infty}\left( V_{DI} \right) \right)\left[ D \right]k_{on}+k_{off}}<\frac{\left[ D \right]}{K_{d}}.$$

Again, for the parameters considered here, $K_{d}=6.8 \mu M$, and at clinical concentrations, the concentration of neutral lidocaine is $\left[ D \right]<8 \mu M$, so $0<\gamma\left( V_{DI} \right)<1.2$. Hence, similar to the dependence on $V_{AP}$, $b^{*}$ is insensitive to shifts in $V_{DI}$ at potentials where steady state inactivation is flat (e.g., $\frac{d\left( 1-h_{\infty}\left( V_{DI} \right) \right)}{dV_{DI}}<1.3\times{10}^{-2} mV^{-1}$, for $V_{DI}<-85 mV$ at $37℃$). However, $b^{*}$ is highly sensitive to shifts in $V_{DI}$ at potentials where $\frac{d\left( 1-h_{\infty}\left( V_{DI} \right) \right)}{dV_{DI}}>0$ (i.e., $V_{DI}>-85 mV$).
