# Supplementary material for: Rate-dependent effects of lidocaine on cardiac dynamics: Development and analysis of a low-dimensional drug-channel interaction model
Source: PLoS Comput Biol. 2021 Jun 29;17(6):e1009145. doi: 10.1371/journal.pcbi.1009145 (PMC8274935; doi:10.1371/journal.pcbi.1009145)
Supplement: S6 Appendix — (DOCX) [file pcbi.1009145.s006.docx]

# Moreno et al. model

The equations for the Moreno et al. [1] Na^+^ current model are provided below. Rate constants with units of $ms^{-1}$ are for $37℃$ and state transition rates are adjusted for changes in temperature using a $Q_{10}$ factor of $3$ (lidocaine binding rates are independent of temperature unless stated explicitly).

## Drug-free Moreno et al. Na^+^ current model:

$$IS=1-\left( O+C1+C2+C3+IC3+IC2+IF \right)$$

$$\frac{dO}{dt}=\beta x\cdot IS+\alpha13\cdot C1+\beta2\cdot IF -\left( \alpha x+\beta13+\alpha2 \right)\cdot O$$

$$\frac{dC1}{dt}=\beta13\cdot O+\alpha3\cdot IF+\alpha12\cdot C2 -\left( \alpha13+\beta3+\beta12 \right)\cdot C1$$

$$\frac{dC2}{dt}=\beta12\cdot C1+\alpha3\cdot IC2+\alpha11\cdot C3 -\left( \alpha12+\beta3+\beta11 \right)\cdot C2$$

$$\frac{dC3}{dt}=\beta11\cdot C2+\alpha3\cdot IC3 -\left( \alpha11+\beta3 \right)\cdot C3$$

$$\frac{dIC3}{dt}=\beta3\cdot C3+\beta11\cdot IC2 -\left( \alpha3+\alpha11 \right)\cdot IC3$$

$$\frac{dIC2}{dt}=\alpha11\cdot IC3+\beta3\cdot C2+\beta12\cdot IF -\left( \beta11+\alpha3+\alpha12 \right)\cdot IC2$$

$$\frac{dIF}{dt}=\alpha12\cdot IC2+\beta3\cdot C1+\alpha2\cdot O -\left( \beta12+\alpha3+\beta2 \right)\cdot IF$$

with rate constants

$$\alpha11=\frac{8.5539}{\left( 7.4392\times{10}^{-2} \right)e^{\frac{-V}{17.0}}+\left( 2.0373\times{10}^{-1} \right)e^{\frac{-V}{150}}}$$

$$\alpha12=\frac{8.5539}{\left( 7.4392\times{10}^{-2} \right)e^{\frac{-V}{15.0}}+ \left( 2.0373\times{10}^{-1} \right)e^{\frac{-V}{150}}}$$

$$\alpha13=\frac{8.5539}{\left( 7.4392\times{10}^{-2} \right)e^{\frac{-V}{12.0}}+ \left( 2.0373\times{10}^{-1} \right)e^{\frac{-V}{150}}}$$

$$\beta11=\left( 7.5215\times{10}^{-2} \right)e^{\frac{-V}{20.3}}$$

$$\beta12=2.7574e^{\frac{-\left( V-5 \right)}{20.3}}$$

$$\beta13=\left( 4.7755\times{10}^{-1} \right)*e^{\frac{-\left( V-10 \right)}{20.3}}$$

$$\alpha3 = \left( 5.1458\times{10}^{-6} \right)e^{\frac{-V}{8.2471}}$$

$$\beta3=6.1205e^{\frac{V}{13.542}}$$

$$\alpha2= 13.370e^{\frac{V}{43.749}}$$

$$\beta2=\frac{\alpha13*\alpha2*\alpha3}{\beta13*\beta3}$$

$$\alpha x = \left( 3.4229\times{10}^{-2} \right)\alpha2$$

$$\beta x = \left( 1.7898\times{10}^{-2} \right)\alpha3$$

## Moreno et al. model of lidocaine-Na^+^ channel interaction:

Non-drug-bound states:

$$IS=1-\left( O+C1+C2+C3+IC3+IC2+IF+D^{+}O+D^{+}C1+D^{+}C2+D^{+}C3+D^{+}IC3+D^{+}IC2+D^{+}IF+D^{+}IS+DO+DC1+DC2+DC3+DIC3+DIC2+DIF+DIS \right)$$

$$\frac{dO}{dt}=\beta x\cdot IS+\alpha13\cdot C1+\beta2\cdot IF+koff\cdot D^{+}O+k\_off\cdot DO-\left( \alpha x+\beta13+\alpha2+kon+k\_on \right)\cdot O$$

$$\frac{dC1}{dt}=\beta13\cdot O+\alpha3\cdot IF+\alpha12\cdot C2+kcoff\cdot D^{+}C1+kc\_off\cdot DC1-\left( \alpha13+\beta3+\beta12+kcon+kc\_on \right)\cdot C1$$

$$\frac{dC2}{dt}=\beta12\cdot C1+\alpha3\cdot IC2+\alpha11\cdot C3+kcoff\cdot D^{+}C2+kc\_off\cdot DC2-\left( \alpha12+\beta3+\beta11+kcon+kc\_on \right)\cdot C2$$

$$\frac{dC3}{dt}=\beta11\cdot C2+\alpha3\cdot IC3+kcoff\cdot D^{+}C3+kc\_off\cdot DC3-\left( \alpha11+\beta3+kcon+kc\_on \right)\cdot C3$$

$$\frac{dIC3}{dt}=\beta3\cdot C3+\beta11\cdot IC2+ki\_off\cdot DIC3-\left( \alpha3+\alpha11+ki\_on \right)\cdot IC3$$

$$\frac{dIC2}{dt}=\alpha11\cdot IC3+\beta3\cdot C2+\beta12\cdot IF+ki\_off\cdot DIC2-\left( \beta11+\alpha3+\alpha12+ki\_on \right)\cdot IC2$$

$$\frac{dIF}{dt}=\alpha12\cdot IC2+\beta3\cdot C1+\alpha2\cdot O+ki\_off\cdot DIF-\left( \beta12+\alpha3+\beta2+ki\_on \right)\cdot IF$$

Charged drug-bound states:

$$\frac{dD^{+}O}{dt}=\beta x1\cdot D^{+}IS+\alpha13c\cdot D^{+}C1+\beta22\cdot D^{+}IF+kon\cdot O-\left( \alpha x1+\beta13c+\alpha22+koff \right)\cdot D^{+}O$$

$$\frac{dD^{+}C1}{dt}=\beta13c\cdot D^{+}O+\alpha33\cdot D^{+}IF+\alpha12\cdot D^{+}C2 +kcon\cdot C1-\left( \alpha13c+\beta33+\beta12+kcoff \right)\cdot D^{+}C1$$

$$\frac{dD^{+}C2}{dt}=\beta12\cdot D^{+}C1+\alpha33\cdot D^{+}IC2+\alpha11\cdot D^{+}C3 +kcon\cdot C2-\left( \alpha12+\beta33+\beta11+kcoff \right)\cdot D^{+}C2$$

$$\frac{dD^{+}C3}{dt}=\beta11\cdot D^{+}C2+\alpha33\cdot D^{+}IC3 +kcon\cdot C3-\left( \alpha11+\beta33+kcoff \right)\cdot D^{+}C3$$

$$\frac{dD^{+}IC3}{dt}=\beta33\cdot D^{+}C3+\beta11\cdot D^{+}IC2 -\left( \alpha33+\alpha11 \right)\cdot D^{+}IC3$$

$$\frac{dD^{+}IC2}{dt}=\alpha11\cdot D^{+}IC3+\beta33\cdot D^{+}C2+\beta12\cdot D^{+}IF -\left( \beta11+\alpha33+\alpha12 \right)\cdot D^{+}IC2$$

$$\frac{dD^{+}IF}{dt}=\alpha12\cdot D^{+}IC2+\beta33\cdot D^{+}C1+\alpha22\cdot D^{+}O -\left( \beta12+\alpha33+\beta22 \right)\cdot D^{+}IF$$

$$\frac{dD^{+}IS}{dt}=\alpha x1\cdot D^{+}O-\beta x1\cdot D^{+}IS$$

with rate constants

$$kon=kcon=\left[ D^{+} \right]500 M^{-1}$$

$$koff=kcoff=500*\left( 318\times{10}^{-6} \right)e^{\frac{-0.7VF}{RT}}$$

$$\alpha x1=\left( 6.3992\times{10}^{-7} \right)\alpha x$$

$$\beta x1=1.3511\beta x$$

$$\alpha13c=\left( 5.6974\times{10}^{-3} \right)\alpha13$$

$$\beta13c=\frac{\beta13*kcon*koff*\alpha13c}{kon*kcoff*\alpha13}$$

$$\alpha22=\left( 6.7067\times{10}^{-6} \right)\alpha2$$

$$\beta22=\frac{\alpha13c*\alpha22*\alpha33}{\beta13c*\beta33}$$

$$\beta33=\left( 1.9698\times{10}^{-5} \right)\beta3$$

$$\alpha33=3.2976\alpha3$$

where $\left[ D^{+} \right]$ is charged drug concentration in $M$, $R=8314.472 mJ/mol\cdot K$ is the gas constant, $F=96485.3415 C/mol$ is the Faraday constant, and $T$ is temperature in Kelvin.

Neutral drug-bound states:

$$\frac{dDO}{dt}=\beta x2\cdot DIS+\alpha\_13\cdot DC1+\beta\_22\cdot DIF+k\_on\cdot O-\left( \alpha x2+\beta\_13+\alpha\_22+k\_off \right)\cdot DO$$

$$\frac{dDC1}{dt}=\beta\_13\cdot DO+\alpha\_33\cdot DIF+\alpha12\cdot DC2 +kc\_on\cdot C1-\left( \alpha\_13+\beta\_33+\beta12+k\_off \right)\cdot DC1$$

$$\frac{dDC2}{dt}=\beta12\cdot DC1+\alpha\_33\cdot DIC2+\alpha11\cdot DC3 +kc\_on\cdot C2-\left( \alpha12+\beta\_33+\beta11+kc\_off \right)\cdot DC2$$

$$\frac{dDC3}{dt}=\beta11\cdot DC2+\alpha\_33\cdot DIC3 +kc\_on\cdot C3-\left( \alpha11+\beta\_33+kc\_off \right)\cdot DC3$$

$$\frac{dDIC3}{dt}=\beta\_33\cdot DC3+\beta11\cdot DIC2 +ki\_on\cdot IC3-\left( \alpha\_33+\alpha11+ki\_off \right)\cdot DIC3$$

$$\frac{dDIC2}{dt}=\alpha11\cdot DIC3+\beta\_33\cdot DC2+\beta12\cdot DIF +ki\_on\cdot IC2-\left( \beta11+\alpha\_33+\alpha12+ki\_off \right)\cdot DIC2$$

$$\frac{dDIF}{dt}=\alpha12\cdot DIC2+\beta\_33\cdot DC1+\alpha\_22\cdot DO+ki\_on\cdot IF-\left( \beta12+\alpha\_33+\beta\_22+ki\_off \right)\cdot DIF$$

$$\frac{dDIS}{dt}=\alpha x2\cdot DO+ki\_on\cdot IS-\left( \beta x2+ki\_off \right)\cdot DIS$$

with rate constants

$$k\_on=\left[ D \right]500 M^{-1}$$

$$k\_off=500\left( 400\times{10}^{-6} \right)$$

$$ki\_on=k\_on/2$$

$$ki\_off=500\left( 3.4\times{10}^{-6} \right)$$

$$kc\_on=k\_on/2$$

$$kc\_off=500\left( 900\times{10}^{-6} \right)$$

$$\alpha x2=\left( 1.3110\times{10}^{-1} \right)\alpha x$$

$$\beta x2=\frac{\beta x*k\_on*\alpha x2*ki\_off}{\alpha x*ki\_on*k\_off}$$

$$\alpha\_13=\left( 8.4559\times10 \right)\alpha13$$

$$\beta\_13=\frac{\beta13*kc\_on*\alpha13n*k\_off}{kc\_off*\alpha13*k\_on}$$

$$\alpha\_22=\left( 1.7084\times{10}^{-5} \right)\alpha2$$

$$\beta\_22=\frac{\alpha\_33*\alpha\_13*\alpha\_22}{\beta\_33 *\beta\_13}$$

$$\beta\_33=4.8477\beta3$$

$$\alpha\_33=\frac{ki\_off*\alpha3*kc\_on*\beta_{33}}{ki\_on*kc\_off*\beta3}$$

where $\left[ D \right]$ is neutral drug concentration in $M$.

# References

1. Moreno JD, Zhu ZI, Yang PC, Bankston JR, Jeng MT, Kang C, et al. A computational model to predict the effects of class I anti-arrhythmic drugs on ventricular rhythms. Sci Transl Med. 2011;3(98):98ra83. doi: 10.1126/scitranslmed.3002588. PubMed PMID: 21885405; PubMed Central PMCID: PMCPMC3328405.
