# Supplementary material for: Rate-dependent effects of lidocaine on cardiac dynamics: Development and analysis of a low-dimensional drug-channel interaction model
Source: PLoS Comput Biol. 2021 Jun 29;17(6):e1009145. doi: 10.1371/journal.pcbi.1009145 (PMC8274935; doi:10.1371/journal.pcbi.1009145)
Supplement: S7 Appendix — (DOCX) [file pcbi.1009145.s007.docx]

# Equations of the low-dimensional lidocaine-Na^+^ channel interaction model

$$I_{Na}=\bar{g}_{Na}m^{3}h\left( 1-b \right)\left( V-E_{Na} \right)$$

$$\frac{dm}{dt}=\alpha_{m}\left( 1-m \right)-\beta_{m}m$$

$$\frac{dh}{dt}=\alpha_{h}\left( 1-h \right)-\beta_{h}h$$

$$\frac{db}{dt}=\left[ D \right]k_{on}\left( 1-h \right)\left( 1-b \right)-k_{off}b,$$

where maximal conductance per capacitance is $G_{Na}=\frac{\bar{g}_{Na}}{C_{m}}=20 nS/pF$ (units as in ten Tusscher et al. [1,2] with $C_{m}$ being membrane capacitance), $k_{on}=250$, $k_{off}=1.7 \times{10}^{-3}$, $\left[ D \right]$ is the concentration of neutral lidocaine,

$$\alpha_{m}=8.743e^{\frac{V}{13.78}}, \beta_{m}=0.1276e^{\frac{V}{-23.25}},\alpha_{h}=\left( 1.187\times{10}^{-5} \right)e^{\frac{V}{-9.328}}, \beta_{h}=2.723e^{\frac{V}{14.91}}$$

at $22℃$. The reversal potential $E_{Na}=\frac{RT}{F}\ln\frac{\left[ Na^{+} \right]_{out}}{\left[ Na^{+} \right]_{in}}$, where $R=8314.472 mJ/mol\cdot K$ is the gas constant, $F=96485.3415 C/mol$ is the Faraday constant, $T$ is temperature in Kelvin, and $\left[ Na^{+} \right]_{out}$ and $\left[ Na^{+} \right]_{in}$ are extracellular and intracellular Na^+^ concentrations, respectively. Units of variables and parameters are $V$ in $mV$, $t$ in $ms$, $k_{on}$ in $M^{-1}ms^{-1}$, $k_{off}$ in $ms^{-1}$, $[D]$ in $M$ and transition rates ($\alpha$’s and $\beta$’s) in $ms^{-1}$. State transition rates are from fitting to drug-free voltage-clamp data in Results 3.1.1 of the main text, and drug binding rates $k_{on}$ and $k_{off}$ are taken from literature [3-5].

Using a $Q_{10}$ factor of $3$, state transition rates were also adjusted for $37℃$

$$\alpha_{m}=45.43e^{\frac{V}{13.78}}, \beta_{m}=0.6628e^{\frac{V}{-23.25}},\alpha_{h}=\left( 6.169\times{10}^{-5} \right)e^{\frac{V}{-9.328}}, \beta_{h}=14.15e^{\frac{V}{14.91}},$$

but lidocaine binding rates are unchanged (as is the case for neutral lidocaine binding rates in the Moreno et al. model [3]).

# References

1. ten Tusscher KH, Noble D, Noble PJ, Panfilov AV. A model for human ventricular tissue. American journal of physiology Heart and circulatory physiology. 2004;286(4):H1573-89. Epub 2003/12/06. doi: 10.1152/ajpheart.00794.2003. PubMed PMID: 14656705.

2. ten Tusscher KH, Panfilov AV. Alternans and spiral breakup in a human ventricular tissue model. American journal of physiology Heart and circulatory physiology. 2006;291(3):H1088-100. Epub 2006/03/28. doi: 10.1152/ajpheart.00109.2006. PubMed PMID: 16565318.

3. Moreno JD, Zhu ZI, Yang PC, Bankston JR, Jeng MT, Kang C, et al. A computational model to predict the effects of class I anti-arrhythmic drugs on ventricular rhythms. Sci Transl Med. 2011;3(98):98ra83. doi: 10.1126/scitranslmed.3002588. PubMed PMID: 21885405; PubMed Central PMCID: PMCPMC3328405.

4. Bennett PB, Valenzuela C, Chen LQ, Kallen RG. On the molecular nature of the lidocaine receptor of cardiac Na+ channels. Modification of block by alterations in the alpha-subunit III-IV interdomain. Circ Res. 1995;77(3):584-92. Epub 1995/09/01. PubMed PMID: 7641328.

5. Liu H, Atkins J, Kass RS. Common molecular determinants of flecainide and lidocaine block of heart Na+ channels: evidence from experiments with neutral and quaternary flecainide analogues. J Gen Physiol. 2003;121(3):199-214. PubMed PMID: 12601084; PubMed Central PMCID: PMCPMC2217334.
