# Supplementary material for: Rate-dependent effects of lidocaine on cardiac dynamics: Development and analysis of a low-dimensional drug-channel interaction model
Source: PLoS Comput Biol. 2021 Jun 29;17(6):e1009145. doi: 10.1371/journal.pcbi.1009145 (PMC8274935; doi:10.1371/journal.pcbi.1009145)
Supplement: S8 Appendix — (DOCX) [file pcbi.1009145.s008.docx]

# Numerical Methods

To simulate our low-dimensional Na^+^ current model, we use two methods to solve the equations for m and h. When simulating voltage-clamp protocols, the solutions for *m* and *h* can be solved explicitly for each period of constant voltage, as the rate constants α_m_, β_m_, α_h_ and β_h_ are constant [1]. However for simulations in which voltage is not fixed, we use the Rush-Larsen scheme to numerically solve for *m* and *h* [2]. Unless otherwise stated, the Rush-Larsen scheme is used to solve for the dynamics of *b*, such that *h* is assumed to be constant over the course of each time step [2]. For voltage-clamp simulations, a time step size of 0.01 *ms* is used to solve the dynamics of *b*.

To numerically solve the Moreno et al. model during voltage-clamp experiments, we utilized the ode15s algorithm from MATLAB 2018b. For single cell and tissue simulations, we used the implicit Trapezoidal method used in the original Moreno et al. 2011 paper [3].

For the single cell and tissue simulations of Section 3.2.1 of the main text, other than the Na^+^ current models described above, all variables in the modified ten Tusscher et al. models are solved using either Rush-Larsen or Forward Euler. Time step sizes of 0.01 *ms* and 0.001 *ms*, respectively, are used for single cell and tissue simulations, respectively. The spatial step size used in tissue simulations is 0.01 *cm*. Reducing the time step size to 0.005 *ms* in single cell simulations results in less than a 1.2% change in peak upstroke velocity estimates, indicating we are at convergence.

# References

1. Teed ZR, Silva JR. A computationally efficient algorithm for fitting ion channel parameters. MethodsX. 2016;3:577-88. doi: 10.1016/j.mex.2016.11.001. PubMed PMID: 27924282; PubMed Central PMCID: PMCPMC5128735.

2. Rush S, Larsen H. A practical algorithm for solving dynamic membrane equations. IEEE Trans Biomed Eng. 1978;25(4):389-92. Epub 1978/07/01. doi: 10.1109/TBME.1978.326270. PubMed PMID: 689699.

3. Moreno JD, Zhu ZI, Yang PC, Bankston JR, Jeng MT, Kang C, et al. A computational model to predict the effects of class I anti-arrhythmic drugs on ventricular rhythms. Sci Transl Med. 2011;3(98):98ra83. doi: 10.1126/scitranslmed.3002588. PubMed PMID: 21885405; PubMed Central PMCID: PMCPMC3328405.
